# The CRISPR ancillary effector Can2 is a dual-specificity nuclease potentiating type III CRISPR defence

**DOI:** 10.1093/nar/gkab073

**Published:** 2021-02-15

**Authors:** Wenlong Zhu, Stuart McQuarrie, Sabine Grüschow, Stephen A McMahon, Shirley Graham, Tracey M Gloster, Malcolm F White

**Affiliations:** Biomedical Sciences Research Complex, School of Biology, University of St Andrews, St Andrews KY16 9ST, UK; Biomedical Sciences Research Complex, School of Biology, University of St Andrews, St Andrews KY16 9ST, UK; Biomedical Sciences Research Complex, School of Biology, University of St Andrews, St Andrews KY16 9ST, UK; Biomedical Sciences Research Complex, School of Biology, University of St Andrews, St Andrews KY16 9ST, UK; Biomedical Sciences Research Complex, School of Biology, University of St Andrews, St Andrews KY16 9ST, UK; Biomedical Sciences Research Complex, School of Biology, University of St Andrews, St Andrews KY16 9ST, UK; Biomedical Sciences Research Complex, School of Biology, University of St Andrews, St Andrews KY16 9ST, UK

## Abstract

Cells and organisms have a wide range of mechanisms to defend against infection by viruses and other mobile genetic elements (MGE). Type III CRISPR systems detect foreign RNA and typically generate cyclic oligoadenylate (cOA) second messengers that bind to ancillary proteins with CARF (CRISPR associated Rossman fold) domains. This results in the activation of fused effector domains for antiviral defence. The best characterised CARF family effectors are the Csm6/Csx1 ribonucleases and DNA nickase Can1. Here we investigate a widely distributed CARF family effector with a nuclease domain, which we name Can2 (CRISPR ancillary nuclease 2). Can2 is activated by cyclic tetra-adenylate (cA_4_) and displays both DNase and RNase activity, providing effective immunity against plasmid transformation and bacteriophage infection in *Escherichia coli*. The structure of Can2 in complex with cA_4_ suggests a mechanism for the cA_4_-mediated activation of the enzyme, whereby an active site cleft is exposed on binding the activator. These findings extend our understanding of type III CRISPR cOA signalling and effector function.

## INTRODUCTION

CRISPR systems provide many bacteria and most archaea with adaptive immunity against mobile genetic elements (MGE) (1–3). They are divided into two distinct classes based on the composition of the effector modules. Class 1 systems (types I, III IV) use multi-subunit effector complexes. Class 2 systems consist of three subtypes (types II, V and VI) and their effector modules are single, large and multifunctional proteins (2).

**Figure 1.**
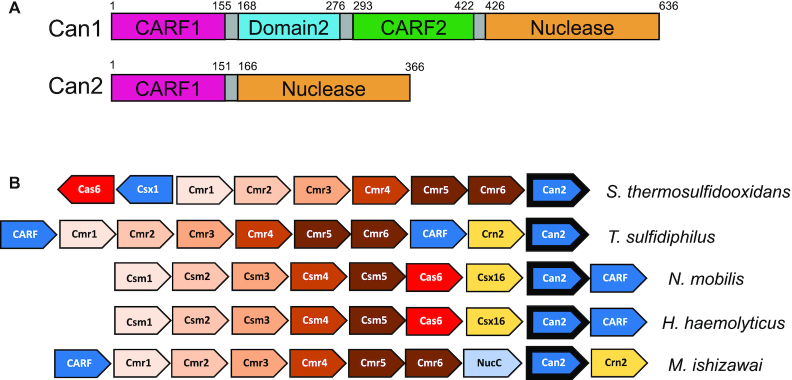
Genome context and domain organisation of Can2. (**A**) Domain organisation of Can1 and Can2. Each has an N-terminal CARF domain and a C-terminal PD-ExK superfamily nuclease domain. The *can1* gene has been predicted to arise from a gene duplication of *can2* (19) and includes Domain 2 – a divergent inactive nuclease domain. The *can2* gene numbering is from *S. thermosulfidooxidans*. (**B**) Gene organisation of selected CRISPR type III systems that include a *can2* gene. Can2 is found associated with both type III-A (Csm) and type III-B (Cmr) systems, and frequently with other CARF family effector proteins. A common neighbouring gene encodes the Crn2 ring nuclease for degradation of cA_4_ (27). Genes labelled as ‘CARF’ encode uncharacterised CARF family proteins. Genes are coloured consistently across the different genomes. Species represented are *Sulfobacillus thermosulfidooxidans (Sth)*, *Thioalkalivibrio sufidiphilus (Tsu)*, *Nitrococcus mobilis*, *Haemophilus haemolyticus* and *Methylomagnum ishizawai*.

Type III CRISPR systems are further divided into Csm (type III-A and III-D) and Cmr (type III-B and III-C) CRISPR systems (2). Their effector complexes are elaborate multi-functional proteins, targeting and cleaving foreign RNA via base-pairing with crRNA (4–6). Target RNA binding in turn can activate two further enzymatic activities within the complex: sequence-nonspecific single-stranded DNA degradation by an HD nuclease domain (7–9) and cyclic oligoadenylate (cOA) synthesis from ATP by a cyclase domain (10–12). In some type III systems, either one or the other of these active sites is absent or non-functional (13–15). cOA is an anti-viral second messenger that activates ancillary effector proteins to potentiate the immune response. To date, several dimeric Csx1/Csm6 family nucleases have been characterised as CRISPR ancillary proteins allosterically activated by cOA binding in the CRISPR associated Rossman fold (CARF) domain within Csx1/Csm6 (16–18). They function as non-specific RNases through their C-terminal HEPN (Higher Eukaryotes and Prokaryotes, Nucleotide binding) domains. *Sulfolobus islandicus* Csx1 forms a trimer of dimers and each dimer binds a cyclic tetra-adenylate (cA_4_) molecule, resulting in non-specific ssRNA cleavage by the C-terminal HEPN domain (16). Moreover, a novel CRISPR defence DNA endonuclease, Can1 (CRISPR ancillary nuclease 1) was identified in our previous study (19). Can1 has a unique monomeric structure with two non-identical CARF domains (Figure 1A). Upon cA_4_ binding in the CARF domain, Can1 rapidly nicks supercoiled DNA non-specifically followed by slower DNA degradation *in vitro* through its C-terminal metal-dependent nuclease domain. Notably, another DNA endonuclease, NucC, is activated by a cyclic tri-nucleotide molecule in response to bacteriophage infection in CBASS (cyclic oligonucleotide-based anti-phage signalling systems) (20,21). NucC homologues have also been identified in association with type III CRISPR systems where they are assumed to be regulated by cyclic tri-adenylate (cA_3_) generated by the Cas10 subunit (20).

Although cOA synthesis is shut off after target RNA cleavage and dissociation from the type III effector complex, activated CRISPR ancillary proteins continue to degrade both viral and cellular nucleic acid (10,22), suggesting that a mechanism for cOA removal might be required. Recently, novel groups of CARF domain proteins, named CRISPR associated ring nuclease 1 (Crn1) (23) and Crn3 (24) were identified, which are dedicated to degrading cA_4_. Moreover, some Csm6 proteins are able to degrade their own cOA activator using their CARF domains (17,18,25). Therefore, various catalytic activities of CARF domain family proteins play important roles in the immune response against MGEs for bacteria and archaea.

In our previous study, we analysed the structure and mechanism of the CARF domain family protein Can1 (19). By comparing the structure of the two halves of Can1 with the DUF1887 family protein VC1899 (PDB: 1XMX) from *Vibrio cholerae*, we postulated that Can1 was derived from an ancestral DUF1887 protein by gene duplication, fusion and subsequent divergent evolution (19). Given the relationship with Can1, DUF1887 family members consisting of a single N-terminal CARF domain fused to a C-terminal nuclease domain will be referred to hereafter as Can2 (CRISPR ancillary nuclease 2). *can2* is much more widely distributed throughout the bacterial phyla than *can1*, particularly in the firmicutes and proteobacteria, and is typically associated with type III CRISPR systems. A selection of type III CRISPR loci incorporating *can2* genes is shown in Figure 1B. *can2* is found in association with genes encoding CARF-family proteins known or predicted to bind cOA (26), and in one case the nuclease NucC. Genes encoding the ring nuclease Crn2, which degrades cA_4_ to deactivate the defence system (27), are found adjacent to *can2* in some genomes, and in others *crn2* is replaced by the uncharacterised *csx16* gene, implicating the latter as a potential ring nuclease (28).

Here, we describe the structure and mechanism of the Can2 protein. We show that Can2 is a metal dependent nuclease, non-specifically degrading both supercoiled DNA and ssRNA, once activated by cA_4_ binding. We co-crystallized Can2 with cA_4_ and solved the structure to 2.0 Å resolution, revealing its detailed molecular architecture and mechanism of activation. Furthermore, we demonstrate that Can2 confers immunity in a reconstituted CRISPR system in *Escherichia coli* by interfering with phage infection.

## MATERIALS AND METHODS

### Cloning

For cloning, synthetic genes (g-blocks) encoding SthCan2, TsuCan2 and VC1899 (full sequences are shown in Supplementary Table S1), codon optimised for expression in *Escherichia coli*, were purchased from Integrated DNA Technologies (IDT), Coralville, USA and cloned into the pEHisV5TEV vector between the NcoI and BamHI sites (29). Competent DH5α (*E. coli*) cells were transformed with the construct and sequence integrity was confirmed by sequencing (GATC Biotech, Eurofins Genomics, DE). The plasmids were then transformed into *E. coli* C43 (DE3) cells for protein expression. The inactivated nuclease domain variants, E276A/D278A for SthCan2, E302A/K304A for TsuCan2 and E291A/D293A for VC1899 were expressed from plasmids where the QuikChange Site-Directed Mutagenesis kit was used to introduce mutations in the wild type genes as per manufacturer's instructions (Agilent Technologies; primers used for mutagenesis are shown in Supplementary Table S2).

### Protein production and purification

For protein expression, the cells expressing each Can2 orthologue in LB medium were grown at 37°C to an OD_600_ of ∼0.8, and then expression was induced with 0.4 mM isopropyl-β-d-1-thiogalactoside (IPTG) and grown overnight at 25°C. Cells were harvested by centrifugation at 3063 × *g* at 4°C for 15 min and resuspended in buffer containing 50 mM Tris–HCl pH 7.5, 500 mM NaCl, 10 mM imidazole and 10% glycerol. A protease inhibitor tablet (Roche; one tablet per 100 ml) and lysozyme (Sigma-Aldrich; 1 mg/ml) were added to the cell suspension, and cells were lysed by sonicating six times for 1 min on ice with 1 min rest intervals. The lysate was cleared at 117 734 × *g* at 4°C for 45 min and loaded onto a pre-equilibrated 5 ml HisTrap FF crude column (GE Healthcare), washed with 5 column volumes (CV) of wash buffer containing 50 mM Tris–HCl pH 7.5, 500 mM NaCl, 30 mM imidazole and 10% glycerol and eluted with a step gradient (holding at 20% for 4 CV and 50% for 4 CV) of elution buffer containing 50 mM Tris–HCl pH 7.5, 500 mM NaCl, 500 mM imidazole and 10% glycerol. Can2-containing fractions were pooled and concentrated using a 10 kDa molecular mass cut-off centrifugal concentrator (Merck). Tobacco Etch Virus (TEV) protease (1 mg per 10 mg protein) was used to remove the polyhistidine affinity tag while dialysing in wash buffer overnight at room temperature. The protein was isolated from TEV protease by the HisTrap FF crude column. The unbound fraction was collected and buffer-exchanged into a buffer containing 50 mM MES pH 6.5 and 150 mM NaCl using a centrifugal concentrator. Can2 was further purified by size exclusion chromatography (S200 26/60; GE Healthcare) in buffer containing 50 mM MES pH 6.5 and 150 mM NaCl. After concentration, Can2 was aliquoted and frozen at –80°C. The three Can2 homologues, SthCan2, TsuCan2 and VC1899 were purified using the same method. Nuclease variants were purified by the same method as for the respective wild-type proteins.

For seleno-methionine labelled expression of SthCan2, the plasmid containing the *can2* gene was transformed into *E. coli* B834 (DE3) cells. Cells were grown in M9 minimal medium supplemented with Selenomethionine Nutrient Mix (Molecular Dimensions, Newmarket, Suffolk, UK) and 50 mg l^−1^ (l)-selenomethionine (Acros Organics). The protein was purified by the same method described for native SthCan2.

### Plasmid cleavage assays

**Figure 2.**
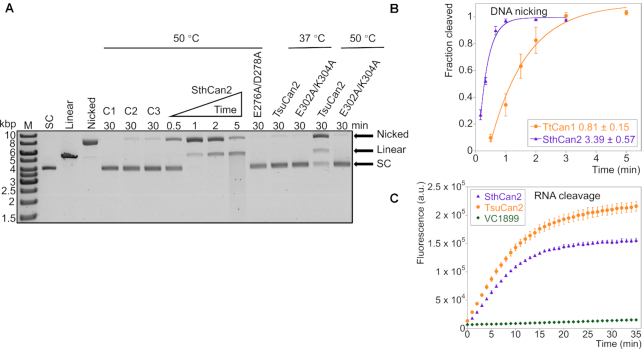
Can2 is activated by cA_4_ to degrade scDNA and RNA. (**A**) Agarose gel analysis of supercoiled plasmid (1.8 nM) nicking and degrading activity by SthCan2 and TsuCan2 (500 nM dimer). Supercoiled plasmid was degraded rapidly by SthCan2 in the presence of cA_4_ (1 μM) and MnCl_2_ (5 mM). Plasmid was incubated with wild-type SthCan2 at 50°C for 0.5, 1, 2 and 5 min in reaction buffer supplemented with cA_4_ and MnCl_2_. The nuclease variant E276A/D278A was incubated under the same conditions for 30 min. Wild-type TsuCan2 and its nuclease variant E302A/K304A were incubated under the same conditions for 30 min at 37 or 50°C. Standards corresponding to supercoiled (SC), linear and nicked plasmid are shown after the marker (M) lane. Control lanes C1, C2 and C3 show the reactions incubated for 30 min without protein, MnCl_2_ and cA_4_, respectively. (**B**) Single-turnover kinetic analysis of scDNA cleavage by SthCan2 and Can1 (the plot for Can1 is from our previous study (19)). SthCan2 (500 nM dimer) was incubated with scDNA (1.8 nM) under the same conditions as in part A and the reaction was stopped at 10 s, 20 s, 40 s, 1 min, 2 min and 3 min. The cleaved fraction of scDNA was plotted against time and fitted to a single exponential curve as described in Materials and Methods. The rate constant of scDNA cleavage for SthCan2 and Can1 are 3.39 ± 0.57 and 0.81 ± 0.15 min^−1^, respectively. Values and error bars represent the mean of triplicate experiments and the standard deviation. (**C**) Plot of fluorescent signals emitted by RNaseAlert substrates when they were cleaved by wild-type SthCan2, TsuCan2 or VC1899. RNaseAlert substrates (30 nM) were incubated with the enzymes (500 nM dimer) in reaction buffer and supplemented with cA_4_ (1 μM) and MnCl_2_ (5 mM) at 37°C. The fluorescent signal was plotted against time. Values and error bars represent the mean of triplicate experiments and the standard deviation.

1.8 nM supercoiled pEV5HisTEV plasmid was incubated with SthCan2 (500 nM dimer; equivalent to 1 μM total protein as measured by absorbance at 280 nm) and its nuclease domain variant E276A/D278A (500 nM dimer) for the time indicated in Figure 2. Reactions were carried out at 50°C in 20 μl final reaction volume at pH 7.0 with the buffer containing 20 mM HEPES, 100 mM NaCl and 1 mM EDTA supplemented with 1 μM cA_4_ and 5 mM MnCl_2_. The standard migration positions for supercoiled, linear or open circle plasmid were ascertained from the incubation with buffer only, with BamHI (Thermo Scientific) or with nicking endonuclease Nt.BspQI (New England BioLabs), respectively. Control reactions included incubating plasmid without protein, MnCl_2_ or cA_4_. For single-turnover kinetics experiments, triplicate experiments were carried by incubating SthCan2 (500 nM dimer) with plasmid substrate (1.8 nM) under the same conditions and stopped at the indicated times by adding 10 mM EDTA. All reactions were analysed by 0.7% agarose gel electrophoresis. Gels were scanned and quantified as described previously (19). Nicked and linearized plasmids are considered as products. This value is divided by the total of products plus substrates, to give the fraction cleaved. The data were plotted against time using Kaleidagraph (Synergy Software) and fitted to a single exponential curve as previously described (19).

To test DNase activity of proteins VC1899 or TsuCan2, 1.8 nM plasmid was incubated with 500 nM dimer protein at 37°C or 50°C in 20 μl final volume in the same buffer as described above. Reactions were supplemented with 1 μM cA_4_ and 5 mM MnCl_2_ as indicated in Supplementary Figure S1. All reactions were stopped by adding 10 mM EDTA and analysed by 0.7% agarose gel electrophoresis.

### RNA cleavage assays

5′-FAM labelled ssRNA (30 nM) was incubated with SthCan2 (500 nM dimer) and E276A/D278A (500 nM dimer) for the desired time. The sequence of the ssRNA is listed in Supplementary Table S2. Reactions were carried out at 50°C in 20 μl final reaction volume in pH 7.0 buffer containing 20 mM HEPES, 100 mM NaCl, 1 mM EDTA and three units SUPERase•In Inhibitor (Thermo Scientific) supplemented with 1 μM cA_4_ and 5 mM MnCl_2_ or MgCl_2_. Control reactions included incubating RNA in buffer only, and incubating RNA without protein, metal ion or cA_4_. Experiments were carried out in triplicate for single-turnover kinetics and quenched by adding to five reaction volumes of phenol chloroform (Ambion) and vortexing. All reactions were analysed by 20% denaturing PAGE (20% acrylamide, 7 M urea and 1× Tris/borate/EDTA (TBE)). Gels were scanned and quantified as described previously (19). The uncleaved ssRNA at each time point was divided by the negative control to give the fraction uncleaved. The fraction cleaved was plotted against the time using Kaleidagraph (Synergy Software) and fitted to a single exponential curve as previously described (19). To test RNase activity of homologous proteins VC1899 or TsuCan1, reactions were carried out at 37°C in the same conditions.

### RNaseAlert fluorimetric assay

RNaseAlert substrates were purchased from Integrated DNA Technologies (IDT). 30 nM substrate was incubated with different enzymes (500 nM dimer) at 37°C for 35 min. Reactions were carried out in a 30 μl final reaction volume in a pH 7.0 buffer containing 20 mM HEPES, 100 mM NaCl, 1 mM EDTA and three units SUPERase•In Inhibitor (Thermo Scientific), supplemented with 1 μM cA_4_ and 5 mM MnCl_2_. The substrates are fluorescence-quenched oligonucleotide probes that emit fluorescence signal after being cleaved. The signal was detected using a microplate reader (FLUOstar Omega, BMG LABTECH) with excitation and emission wavelengths set at 485 and 520 nm, respectively.

### Radiolabelled cA_4_ cleavage assays

^32^P labelled cA_4_ was generated by incubating *S. solfataricus* type III-D complex with α-^32^P-ATP as described previously (30). For cA_4_ cleavage assays, ∼10 nM radiolabelled cA_4_ was incubated with different concentrations of SthCan2. Reactions were carried out at 50°C for 30 min in 20 μl final volume in 20 mM HEPES, 100 mM NaCl, 1 mM EDTA, pH 7.0 and three units SUPERase•In Inhibitor supplemented with 5 mM MgCl_2_. The control reaction was carried out by incubating radiolabelled cA_4_ in buffer only under the same conditions. All reactions were quenched and deproteinized by phenol-chloroform extraction, then chloroform extraction, before loading onto thin-layer chromatography (TLC) plates. Plates were visualized by phosphor imaging.

### Plasmid ligation and supercoiling

1.5 nM pEV5HisTEV plasmid was incubated with SthCan2 (100 nM dimer) at 50°C for 1.5 min in the pH 7.0 buffer described above, supplemented with 200 μM cA_4_ and 5 mM MnCl_2_. Reactions were quenched and deproteinized by PCR Clean-Up System (Promega). The eluted product was incubated with DNA ligase and gyrase as described in a previous study (19). Nicking endonuclease Nt.BspQI was used as a positive control. All reactions were analysed by 0.7% agarose gel electrophoresis.

### Plasmid transformation assay

**Figure 3.**
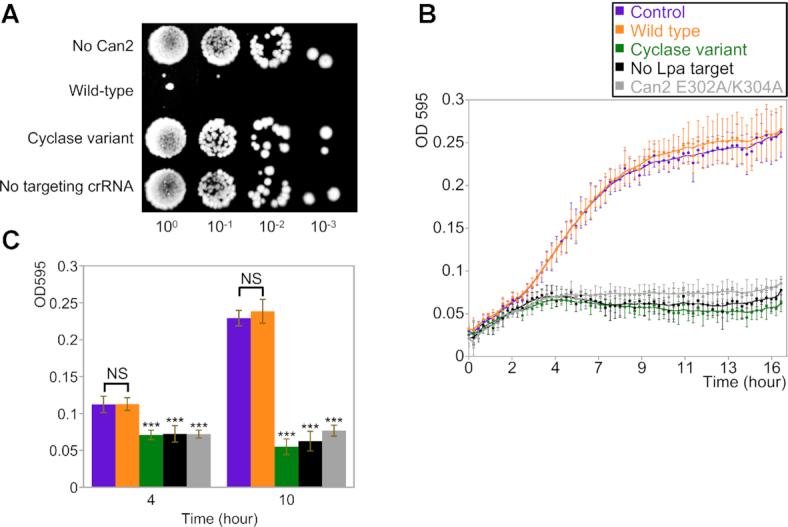
Can2 protects against MGE *in vivo*. (**A**) Plasmid challenge assay of mycobacterial type III-A system in *E. coli* host. *E. coli* cells harbouring the mycobacterial type III-A interference complex Csm1-5 and TetR targeting spacer were transformed with plasmid containing TsuCan2 effector and tetracycline-resistance gene (Wild-type). Other strains are indicated as ‘No Can2’ where the TsuCan2 is absent, ‘Cyclase variant’ where the interference complex is unable to produce cOA and ‘No targeting crRNA’ where the TetR targeting spacer is replaced with a spacer targeting pUC19 MCS. A 10-fold dilution series of the transformation mixture was applied onto tetracycline selective plates to determine the number of viable transformants. (**B**) Growth curves of *E. coli* cells harbouring the interference complex and phage P1 *lpa* gene-targeting spacer supplemented with TsuCan2 effector (indicated as Wild-type). Cells were grown in LB broth in a 96-well plate with shaking at 37°C and infected with phage P1 at a MOI of ∼1. OD_595_ of the culture was measured every 15 min to plot against time over the 16 h incubation. Other strains are indicated as ‘Cyclase variant’ (green) where the interference complex is unable to produce cOA molecules; ‘No Lpa target’ (black) where *lpa* gene-targeting spacer is replaced with a spacer targeting pUC19 (MCS); ‘Can2 E302A/K304A’ (grey) which is a TsuCan2 nuclease variant. ‘Control’ (purple) represents wild-type cells (orange) incubated without phage infection under the same conditions. Data points represent the mean of eight experimental replicates (four biological replicates with two technical replicates each) with the standard deviation shown. (**C**) The OD_595_ values of all strains after 4 and 10 h growth are shown, coloured as in panel B. Statistical analysis was carried out with RStudio using the unpaired Welch two sample test to calculate *P*-values. NS (not significant) indicates *P*-values > 0.05 and *** indicates *P*-values < 1E–05.

The construction of the pCsm1-5_ΔCsm6 plasmid (containing the type III Csm interference genes *cas10* (*csm1*), *csm3*, *csm4*, *csm5* from *M. tuberculosis* and *csm2* from *M. canettii*) and pCsm1-5_Cy plasmid expressing an inactivated cyclase variant (Csm1 D630A/D631A) of the Csm complex has been described previously (15,30). The cyclase variant is unable to synthesise cOA signalling molecules due to mutation of the cyclase domain (15,30). Plasmid pCRISPR_TetR contains four identical spacers targeting the tetracycline-resistance gene and five repeats from *M. tuberculosis* (15,30). Plasmid pCRISPR which consists of two identical spacers targeting the pUC19 multiple cloning site (MCS) and three repeat sequences from *M. tuberculosis* was used for the ‘Not targeting crRNA’ control (15,30). Both plasmid pCRISPR_TetR and pCRISPR contain *M. tuberculosis cas6*. Plasmid pRAT containing the tetracycline-resistance gene without insert (i.e. without effector gene) was used as the ‘No Can2’ control (15,30). Plasmid pRAT-Duet containing the tetracycline-resistance gene has been described previously (30). Plasmid pRAT_TsuCan2 was constructed by cloning *can2* from *T. sulfidiphilus* into the 5′-NcoI and 3′-SalI sites of the pRAT-Duet MCS-1 vector by restriction digest. The plasmid transformation assay was carried out essentially as described previously (15,30). *E. coli* C43 (DE3) cells containing pCsm1-5_ ΔCsm6 and pCRISPR_TetR were transformed by heat shock with 50 ng pRAT_TsuCan2 or pRAT plasmid, indicated as ‘Wild-type’ or ‘No Can2’ in Figure 3A, respectively. *E. coli* cells containing pCsm1-5_Cy and pCRISPR_TetR were transformed with 50 ng of pRAT_TsuCan2 plasmid indicated as ‘Cyclase variant’ in Figure 3A. *E. coli* C43 cells containing pCsm1-5_ ΔCsm6 and pCRISPR were transformed with 50 ng of pRAT_TsuCan2 indicated as ‘No targeting crRNA’ in Figure 3A. After outgrowth at 37°C for 2.5 h, 5 μl of a 10-fold dilution series was applied onto LB agar containing 100 μg ml^−1^ ampicillin and 50 μg ml^−1^ spectinomycin to determine the cell density of the recipient cells and onto LB agar additionally containing 25 μg ml^−1^ tetracycline, 0.2% (w/v) d-lactose and 0.2% (w/v) l-arabinose to determine the number of viable transformants. Plates were incubated at 37°C for 40 h. The experiment was carried out with two biological replicates and four experimental replicates each.

### Bacteriophage infection assay

A CRISPR array consisting of three identical spacers (Supplementary Table S2) targeting the *lpa* gene of bacteriophage P1 and four Mtb CRISPR repeats was ligated into the pCDFDuet™-1 vector (Novagen, Merck Millipore) to give pCRISPR_Lpa using the method described previously (15,30). *T. sulfidiphilus* (Tsu) *can2* was cloned into the pEV5HisTEV vector between the NcoI and BamHI sites, and the nuclease domain variant E302A/K304A was generated using the QuikChange Site-Directed Mutagenesis kit as per manufacturer's instructions (Agilent Technologies). Plasmid pRAT_TsuCan2_E302A/K304A was constructed by cloning the variant gene from this pEV5HisTEV construct into the 5′-*Nco*I, 3′-*Sal*I sites of pRAT-Duet MCS-1.

Plasmids pCsm1-5_ ΔCsm6, pCRISPR_Lpa and pRAT_TsuCan2 were co-transformed into *E. coli* C43 (DE3) cells indicated as ‘Wild-type’ in Figure 3B. Plasmids pCsm1-5_Cy, pCRISPR_Lpa and pRAT_TsuCan2 were co-transformed into *E. coli* cells and are indicated as ‘Cyclase variant’ in Figure 3B. Plasmids pCsm1-5_ΔCsm6, pCRISPR and pRAT_TsuCan2 were co-transformed into *E. coli* cells and are indicated as ‘No Lpa target’ in Figure 3B. Plasmids pCsm1-5_ΔCsm6, pCRISPR_Lpa and pRAT_TsuCan2_E302A/K304A were co-transformed into *E. coli* cells and are indicated as ‘Can2 E302A/K304A’ in Figure 3B. The cells were grown overnight at 37°C in LB broth containing 50 μg ml^−1^ ampicillin, 25 μg ml^−1^ spectinomycin and 12.5 μg ml^−1^ tetracycline. The overnight culture was diluted to OD_600_ of ∼0.1 (light path length: 10 mm) by LB broth supplemented with the antibiotics, 10 mM MgSO_4_, 0.2% (w/v) d-lactose and 0.2% (w/v) l-arabinose. 160 μl of diluted culture was infected with 40 μl bacteriophage P1 to give a MOI around 1 and was grown in a 96-well plate. The OD_595_ of the culture in the plate (light path length: ∼6.2 mm) was measured by a FilterMax F5 Multi-Mode Microplate Reader (Molecular Devices) every 15 min over 16 h incubation time. The experiment was carried out with four biological replicates and two technical replicates. The OD_595_ was plotted against time over the 16 h incubation.

### Co-crystallisation of Can2 in complex with cA_4_

SthCan2 labelled with selenomethionine at 8 mg/ml was mixed with cA_4_ such that the molar ratio of protein:cA_4_ was 1:1. Crystallisation conditions were identified from the commercial screens JCSG and PACT 96 (Jena Biosciences). 75 μl of the mother liquor was added to the reservoir in a 96-well sitting drop plate, and the SthCan2 + cA_4_ solution was mixed with mother liquor in a 0.45 μl drop with a 2:1 or 1:1 protein:mother liquor ratio. Plates were incubated at room temperature. Initial screens yielded optimal crystals in 20% (w/v) PEG 3350 and 0.2 M ammonium nitrate pH 6.3, which required no further optimisation prior to data collection. Crystals were harvested into a fresh 1 μl drop of mother liquor and 1 μl glycerol was added to the drop for cryo-protection. Crystals were mounted on loops and vitrified in liquid nitrogen.

### X-ray data processing, structure solution, and refinement

Data were collected at Diamond Light Source (DLS) on beamline I03 at a wavelength of 0.9790–2.02 Å resolution (data processing and refinement statistics are shown in Supplementary Table S3). Diffraction images were automatically processed through the Xia2 pipeline (31) using XDS (32) and AIMLESS (33). Phasing information and structure solution was done using the automated experimental phasing pipeline BigEP (34) at DLS, which used SHELX (35) to assess the quality of the anomalous signal and CRANK (36) for phasing and to build the initial model. REFMAC5 (37) and COOT (38) were used for refinement of the model, and addition of ligands and water molecules. The cA_4_ ligand was drawn using Chemdraw (Perkin Elmer) and restraints generated in JLigand (39). Figures of the structures were created by CCP4mg (40) and PyMol (Schrödinger, LLC). The model was validated using tools in PDB-REDO (41) and Molprobity (42). The final Molprobity score is 1.2, centile 100, and Ramachandran statistics are 98.96% allowed, 0% disallowed. All structural alignment calculations were done using DALI (43). The coordinates and structure factors have been deposited in the Protein DataBank with accession code 7BDV.

### Statistics

Statistical analyses were performed with GraphPad Prism. Statistical significance was assessed as described for each experiment.

## RESULTS

### Can2 is a cA_4_ activated, metal dependent nuclease

To study the structure and mechanism of Can2, we expressed and purified the wild-type Can2 protein from the moderate thermophile *S. thermosulfidooxidans* (SthCan2) along with a E276A/D278A variant, targeting the C-terminal PD-ExK nuclease domain. Both enzymes were purified by immobilised metal affinity and gel filtration chromatography as described in the methods. Wild-type SthCan2 exhibited potent nuclease activity, degrading a supercoiled plasmid substrate (scDNA) after 5 min incubation in the presence of cA_4_ and MnCl_2_ at 50°C (Figure 2A). The majority of the plasmid was nicked within 1 min and after 5 min incubation most of the plasmid was linearized, with some degrading to smaller fragments. Reactions in the absence of Can2, MnCl_2_ or cA_4_ are shown in lanes C1, C2 and C3, respectively, in Figure 2A; they display very little activity, confirming that SthCan2 is a metal dependent nuclease activated by cA_4_. The rate of plasmid degradation for SthCan2 was determined under single turnover conditions, using 500 nM protein dimer incubated with 1.8 nM scDNA, as *k*_c_ = 3.4 ± 0.57 min^−1^ (Figure 2B) which was about 4-fold higher than the rate of the nickase Can1 under similar conditions (19). The E276A/D278A variant was inactive, confirming that Can2 is a PD-ExK superfamily nuclease. This glutamate and aspartate residue form part of the highly conserved active site motif in PD-ExK nucleases, and if mutated abrogate activity. As observed previously for Can1, the nicked DNA products generated by SthCan2 were readily re-ligated by DNA ligase, consistent with generation of 3′-hydroxyl and 5′-phosphate ends (Supplementary Figure S2). Overall, the results suggest SthCan2 has cA_4_-activated DNA nickase activity, as observed previously for Can1 (19).

We also analysed a homologous protein from the mesophile *T. sulfidiphilus* (TsuCan2). TsuCan2 and its nuclease variant E302A/K304A were incubated with scDNA under the same conditions at both 37 and 50°C (Figure 2A and Supplementary Figure S1B). Most scDNA was nicked after 30 min incubation only at 50°C, with markedly slower kinetics than for SthCan2. The E302A/K304A variant was inactive. Another homologue, VC1899 from *Vibrio cholerae*, was not able to nick or degrade scDNA after 30 min incubation at either 37 or 50°C (Supplementary Figure S1A and S1B).

We proceeded to test the RNase activity of the three proteins using the RNaseAlert fluorometric assay (Figure 2C). The substrates were fluorescence-quenched oligonucleotide probes that emit a fluorescent signal after being cleaved, and have been used previously for studies of the Csm6 family enzymes (12,18). After 20 min incubation, the majority of RNaseAlert substrates were degraded by SthCan2 and TsuCan2. In contrast, VC1899 showed very little RNase activity. Controls in the absence of manganese or cA_4_ activator confirmed that the RNase activity was metal and cA_4_ dependent (Supplementary Figure S1C). Little or no RNase activity was observed among any nuclease variants (Supplementary Figure S1D). To confirm the RNase activity of Can2, we tested both enzymes in an assay with a fluorescent RNA substrate (Supplementary Figure S3). SthCan2 cleaved this RNA rapidly in the presence of Mn^2+^, with a rate constant in excess of 5 min^−1^, precluding accurate quantification. When the assays were repeated in the presence of Mg^2+^, the reaction rate was reduced allowing quantification of the RNA cleavage rate for SthCan2 as 1.2 ± 0.13 min^−1^ (Supplementary Figure S3D).

Finally, we tested SthCan2 for the ability to degrade the cA_4_ activator (‘ring nuclease’ activity) in the presence of Mg^2+^ and observed that this activity was not present (Supplementary Figure S4), consistent with previous observations for Can1 (19). Recent studies suggest ring nucleases are widespread (23,24,27,30) and most probably essential if type III CRISPR systems are not functioning in defence by abortive infection (22). The genomic context of the *can2* gene suggests that ring nuclease duty is ‘outsourced’ to dedicated enzymes such as Crn2 and the putative ring nuclease Csx16 (Figure 1).

### Can2 provides immunity against MGE in vivo

To investigate the capability of Can2 to confer immunity against MGE *in vivo*, we utilised a recombinant type III CRISPR system from *Mycobacterium tuberculosis* expressed in *E. coli (**15**)*. We combined plasmid pCsm1-5_ΔCsm6 containing the interference complex module Csm1-5 and plasmid pCRISPR_TetR containing the tetracycline-resistance gene-targeting spacer (TetR targeting spacer) in *E. coli* C43 (DE3) cells, as described in the Methods and previously (15,30). This recombinant CRISPR system was previously confirmed as functional and able to synthesise a range of cyclic oligonucleotides, including cA_3–6_, in the presence of target RNA (15,30). The system allows cA_6_ or cA_4_ dependent downstream effectors to be incorporated and provide effective immunity against a plasmid transformation challenge. We have shown previously that the HD nuclease domain of *M. tuberculosis* Csm is not capable of providing immunity and has little or no activity *in vitro* (15,30). Therefore, we constructed the plasmid pRAT_TsuCan2 which harbours the *can2* gene from *T. sulfidiphilus* (Tsu, chosen as it is a mesophile) and a tetracycline-resistance gene containing a match to the spacer on pCRISPR_TetR.

The *E. coli* cells containing pCsm1-5_ΔCsm6 and pCRISPR_TetR were transformed with pRAT_TsuCan2. If the type III interference complex detects cognate mRNA from the TetR locus, the cyclase domain will be activated to generate cA_4_. A 10-fold dilution series of the transformation mixture was applied to tetracycline selective plates to determine the number of viable transformants (Figure 3A). Negative control strains included were pRAT without TsuCan2 (No Can2); a variant of Cas10 which cannot produce cOA (Cyclase variant); and crRNA where the TetR targeting spacer was replaced with a spacer targeting the pUC19 multiple cloning site (No targeting). Previous studies demonstrated that the *M. tuberculosis* type III-A CRISPR system requires an ancillary nuclease to provide immunity *in vivo (**15**)*. Compared to the negative controls, three orders of magnitude fewer transformants were observed for cells expressing wild-type TsuCan2 (Figure 3A). Thus, in the presence of cA_4_, Can2 provides effective immunity against incoming MGE, similar to the previously characterised Csx1 effector (15).

The plasmid challenge assay cannot discriminate between plasmid clearance and cell death, due to the requirement of tetracycline resistance for growth. We therefore developed a viral infection challenge assay using the temperate bacteriophage P1 (44). We constructed another plasmid, pCRISPR_Lpa, which contains a spacer targeting the *lpa* gene of bacteriophage P1. Plasmids pCsm1-5_ΔCsm6, pCRISPR_Lpa and pRAT_TsuCan2 were co-transformed into *E. coli* C43 (DE3) cells. The cells were grown in LB broth in a 96-well plate with shaking at 37°C and infected with phage P1 at a multiplicity of infection (MOI) of ∼1. Cell growth was monitored by measuring the optical density at a wavelength of 595 nm (OD_595_) (Figure 3B). The control culture (purple) was not infected with the phage and thus grew normally, providing a standard growth curve for the experiment. Cells with defence based on wild-type Can2 (orange) were not affected by phage infection, growing similarly to the control culture. In contrast, a strain where the Lpa-targeting spacer was missing (black) was highly susceptible to phage infection, as was a strain lacking cyclase activity (green), and a strain harbouring the inactive E302A/K304A Can2 variant (grey). The OD_595_ reading of each strain after 4 h and 10 h growth was quantified and is shown in Figure 3C. No significant difference was observed between control cells and cells expressing wild-type Can2 (*P*-values > 0.05), whereas the growth of other strains was significantly reduced compared to the control culture at both 4 h and 10 h (*P*-values < 1E-05). These results show that immunity depends on a targeting crRNA matching the phage, synthesis of cA_4_ by the cyclase, and the nuclease activity of the Can2 effector.

### Structural analysis of Can2

SthCan2, with selenomethionine incorporated for phasing, was co-crystallised with cA_4_. Diffraction data were collected to 2.02 Å resolution at Diamond Light Source, and the data were processed, scaled, phased from the selenium atoms, and the initial model built using their automated pipeline. Subsequent rounds of automated and manual refinement gave a final *R*_work_ of 24% and *R*_free_ of 29%. There are four protein molecules in the asymmetric unit, which form two dimers each bound to a molecule of cA_4_. The protein chain can be traced from residue 3 to 366, although there is variation between protein molecules; some miss residues at the N-terminus and/or three different loop regions (centred around residues 160, 222 and/or 300). Efforts were made to crystallise apo SthCan2, but unfortunately no crystals were forthcoming.

**Figure 4.**
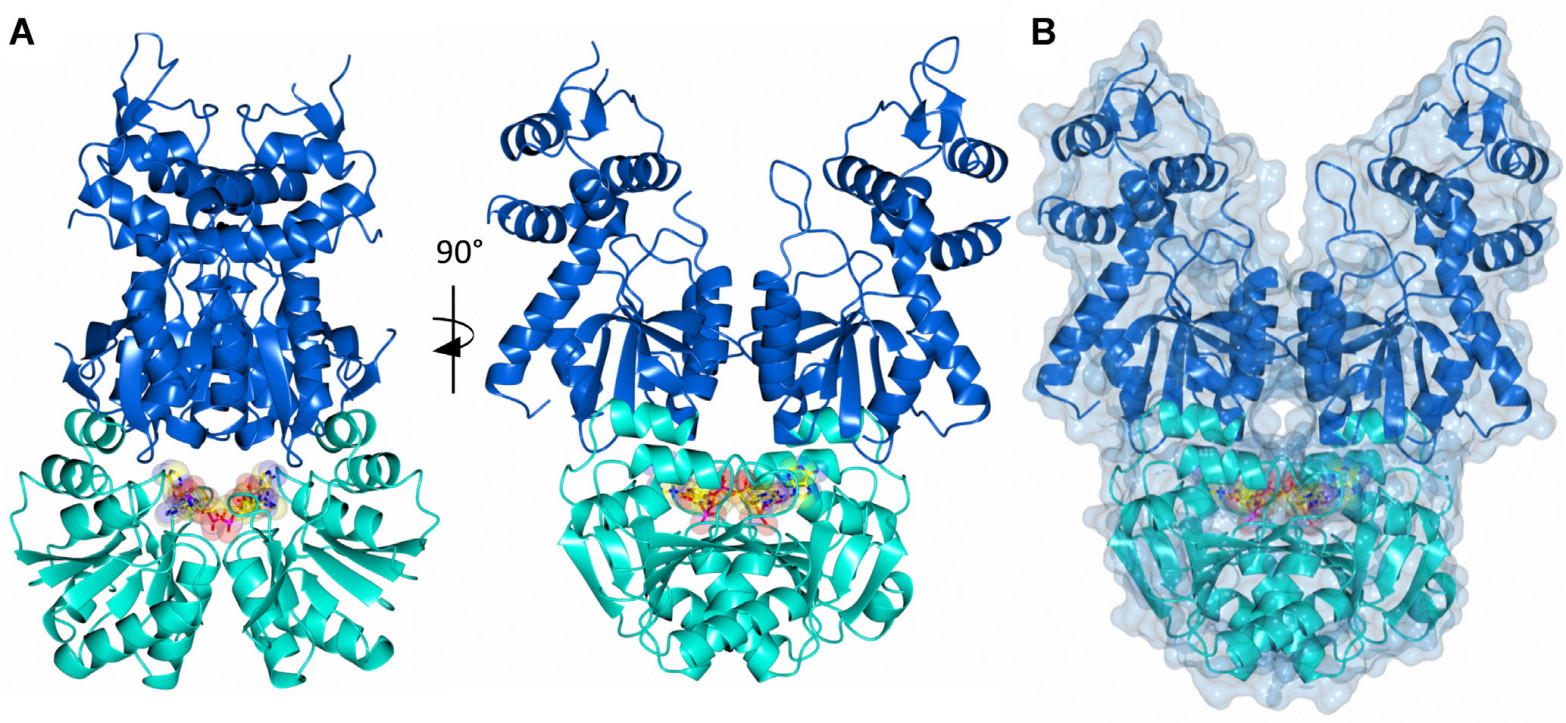
Structure of Can2 bound to cA_4_ activator. (**A**) Two views of the Can2 dimer in cartoon representation. The CARF domain is shown in cyan and the nuclease domain in blue. A molecule of cA_4_ is bound across the CARF dimer, which is shown as spheres (carbon in yellow, oxygen in red, nitrogen in blue, phosphate in magenta). (**B**) Surface representation of Can2 dimer with the same colouring as (A).

**Figure 5.**
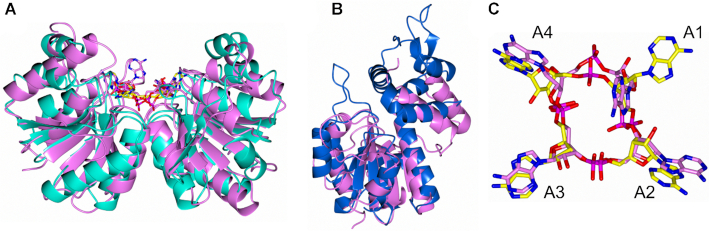
Structural comparison of Can2 with Can1. (**A**) Superimposition of CARF domain dimer from Can2 (cyan) bound to cA_4_ (yellow) with the CARF domains of Can1 bound to cA_4_ (PDB: 6SCE) (pink). The Can2 CARF domain dimer superimposes with the Can1 CARF domains with an RMSD of 2.6 Å over 256 Cα atoms. (**B**) Superimposition of nuclease domain from Can2 (blue) and Can1 (pink) (RMSD of 3.2 Å over 139 Cα atoms). (**C**) Superimposition cA_4_ molecules bound to Can2 (yellow) and Can1 (pink) shown in stick representation (carbon in yellow/pink, oxygen in red, nitrogen in blue, phosphate in magenta; RMSD 3.1 Å over 88 atoms). Each AMP is numbered (A1-A4).

Can2 has two distinct domains, linked by a 15-residue highly flexible loop (Figure 4A, B). At the N-terminus there is a CARF domain (residues 3–151) and at the C-terminus a nuclease domain (residues 166–366). The CARF domain displays the canonical Rossman fold, which is highly structurally conserved with Can1 (PDB: 6SCE; RMSD of 2.1 Å over 137 Cα atoms for Can2 monomer/RMSD of 2.6 Å over 256 Cα atoms for Can2 dimer; Figure 5A) and VC1899 (PDB:1XMX; RMSD of 1.7 Å over 141 Cα atoms; Supplementary Figure S5A). The nuclease domain is typical of the PD-D/ExK metal-dependent nuclease family, which comprises a central core of six β-strands flanked by six α-helices. The nuclease domain of Can2 is highly conserved with VC1899 (RMSD of 2.5 Å over 186 Cα atoms), but slightly less so with Can1 (RMSD of 3.2 Å over 139 Cα atoms; Figure 5B). This is due to a motif additional to the core nuclease fold present in Can2 and VC1899 comprising four α-helices flanking two antiparallel β-strands; in Can 1 a helix-turn-helix motif is in a near equivalent position.

### cA_4_ recognition by Can2

**Figure 6.**
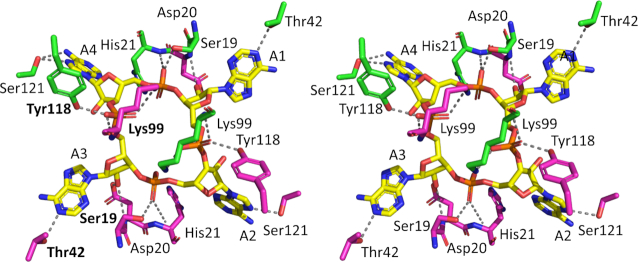
Structural comparison of cA_4_ bound to Can2. Divergent stereo representation of cA_4_ (in stick representation; carbon in yellow, nitrogen in blue, oxygen in red; phosphorus in orange) in complex with the Can2 CARF domain dimer (in stick representation; each monomer in the dimer is coloured green or magenta). Each AMP is numbered (A1–A4). The dotted lines represent hydrogen bond interactions. The residue labels in bold indicate there is a conserved interaction between cA_4_ in both Can 1 and Can2.

Unambiguous electron density in the *F*_obs_ – *F*_calc_ map at 3σ at the interface between the CARF domains in the dimer of Can2 corresponded to a molecule of cA_4_ (Supplementary Figure S6) enclosed in the binding site (Figure 4B). The cA_4_ molecule makes symmetrical interactions with each Can2 monomer, which differs from the asymmetric interactions made between Can1 and cA_4_. All interactions made between Can2 and cA_4_ are solely from the CARF domains (Figures 5A, 6A). Hydrogen bonds form between cA_4_ and main chain residues of Asp20, His21, and Lys99 and side chain residues of Ser19, Asp20, Thr42, Lys99, Tyr118 and Ser121 in both molecules of the Can2 dimer. Superposition of the cA_4_ molecules from the complexes with Can1 and Can2 (RMSD of 3.1 Å over 88 atoms; Figure 5C) show the majority of the atoms are in a similar position. The most significant difference is one of the adenosine moieties (A1, Figure 5C). It was noted that in Can1, the adenine base of A1 adopted an axial position at the anomeric carbon of the ribose, which likely arose from a π-π stacking interaction with a nearby tryptophan residue. In Can2, which lacks this key tryptophan residue, the adenosine in the equivalent position adopts a similar conformation to the others in the cA_4_ molecule.

### Structural comparisons of cA_4_ binding in Can2 with Can1 and VC1899

Although the overall sequence identity between Can2 and Can1 is low, there is a high level of structural conservation (Supplementary Figure S7A). Closer examination of the binding site interactions made between the CARF domains of Can1 and Can2 with cA_4_ highlighted the residues that are likely to be key from an evolutionary standpoint. The interactions formed by just four residues were conserved between the two structures (Figure 6, Supplementary Figure S8A, B); these are (Can2 numbering) Ser19, Thr42, Lys99, and Tyr118. Ser19 and Tyr118 each form a single hydrogen bond with a (different) phosphate group, and Thr42 with an adenine base. The conserved lysine (Lys99) in both Can1 and Can2 forms hydrogen bonds, via both its terminal and main chain nitrogen atoms, with the two non-bonded oxygen atom/hydroxyl group in a single phosphate moiety. What is striking about these four residues is they interact with the same ‘side’ of the cA_4_ molecule (A3 and A4 in Figure 6), even though three of the residues originate from one of the Can2 monomers/second CARF domain of Can1, and one residue from the other Can2 monomer/first CARF domain of Can1. Sequence comparison of Can2 with Can1 shows the C-terminal CARF and nuclease domains of the latter have the highest similarity to Can2, which is consistent with the conservation of the interactions observed. Therefore, it is likely that the C-terminal ‘half’ of Can1 and Can2 are evolutionarily related, and the N-terminal ‘half’ of Can1 arose from gene duplication. This may explain why the Can1 nuclease-like domain lacks the canonical nuclease active site residues, but it is intriguing why the first CARF domain of Can1 has evolved to interact differently with the other ‘half’ of cA_4_ (ie. A1 and A2 in Figure 6).

The highest overall sequence homology and structure conservation for Can2 is with VC1899. Comparison of the cA_4_ binding site (Supplementary Figure S8A, C), however, reveals there is no conservation of residues in VC1899 that interact with cA_4_ in Can2. The four residues that were observed to be conserved between Can1 and Can2, both in sequence and structure, Ser19, Thr42, Lys99 and Tyr118, are Asp9, Asp34, Arg96 and Val115, respectively in VC1899. Other residues in VC1899 surrounding the modelled cA_4_ molecule are Gln10, Asp11, Arg14, Pro118, Leu95 and Gln334. The sole example of conservation of residue properties is Arg96, which is equivalent to Lys99 in Can2. The rest of the predicted binding site residues show different properties to their equivalent residues in Can2, with several possessing a negative charge (Asp9, Asp11, Asp34) or are hydrophobic (Val115, Pro118, Leu95). These properties, especially the residues with a negative charge, would not be conducive to interacting with a negatively charged cA_4_ molecule, and contrasts with the Can2 binding site where all residues are polar and capable of forming hydrogen bond interactions with cA_4_. The nature of the binding site, which would not support binding of cA_4_ without a high energetic barrier, therefore supports the biochemical studies which show that cA_4_ is not capable of activating nuclease activity in VC1899.

### Recognition of DNA by the nuclease domain

**Figure 7.**
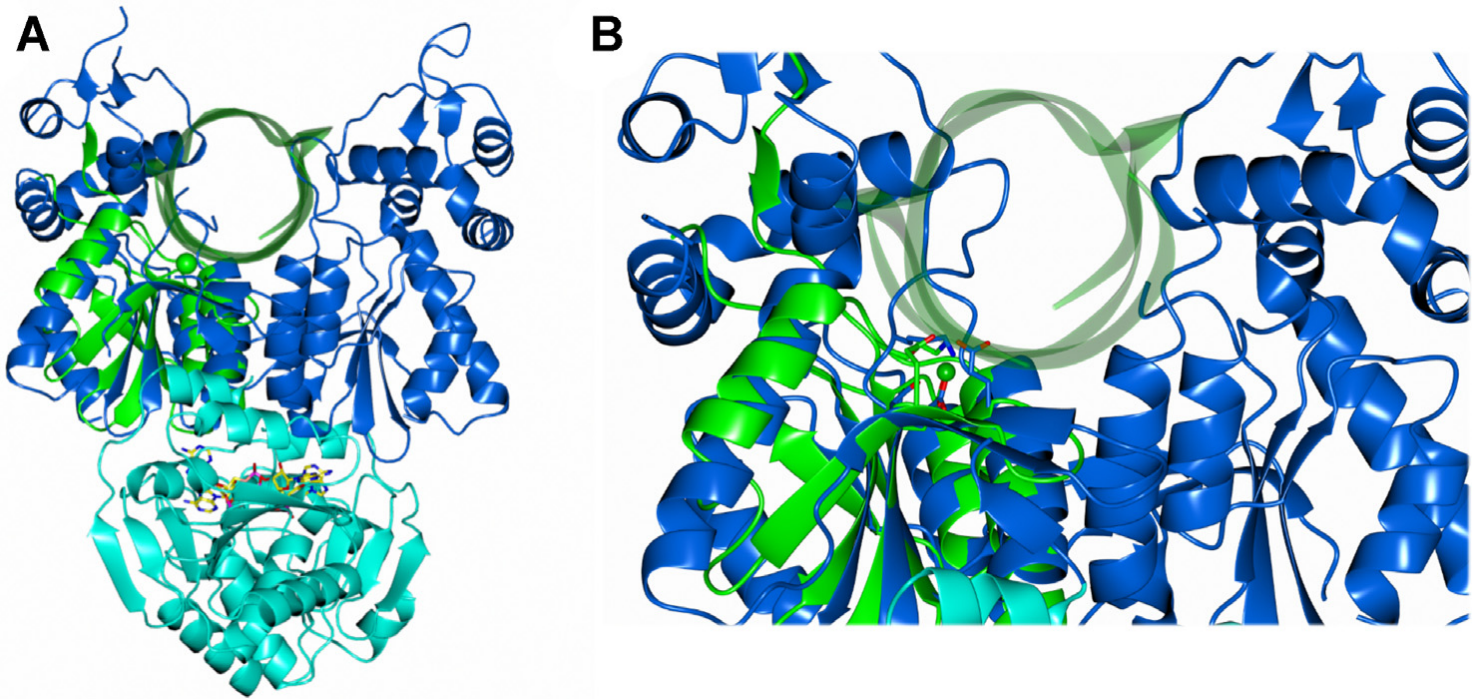
Predicted ternary complex of Can2 and cA_4_, with dsDNA modelled. (**A**) Superimposition of the nuclease domain of Can2 (blue) with the nuclease domain of EndoMS in complex with dsDNA (PDB: 5GKE) in cartoon representation (green) (RMSD 4.4 Å over 108 Cα atoms). (**B**) Close-up view of panel A. The active site residues of Can2 (Glu276 and Asp278) are shown as blue sticks, and the active sites residues (Glu156 and D158A mutant) and metal ion (Mg^2+^) of EndoMS shown as green sticks and sphere respectively.

The structure of Can2 bound to cA_4_ represents the active state of the enzyme. To understand the likely mechanism of the nuclease, and in particular the recognition of nucleic acid substrates, we investigated structural similarities between Can2 and other PD-ExK family nucleases. The closest structural homologue to the nuclease domain of Can2 with dsDNA bound was the mismatch repair enzyme EndoMS (45), a dimeric enzyme that detects mismatches in dsDNA and introduces nicks in both DNA strands, resulting in a double strand break. The nuclease domains of Can2 and EndoMS align with an RMSD of 4.4 Å over 108 Cα atoms, and the secondary structure elements in the core of the nuclease and the active site residues superimpose well (Figure 7A, B; Supplementary Figure S9). A similar comparison with the restriction endonuclease *Age*I (46) confirms the nuclease superfamily fold and conservation of key catalytic residues in a suitable position to engage with DNA (Supplementary Figure S10). These comparisons help to define the site of nucleic acid binding in Can2. The dsDNA substrate from EndoMS in the superimposed structures sits in the cleft between the two Can2 nuclease domains (Figure 7). There appears to be room in this cleft to accommodate canonical dsDNA if it is assumed that the flexible loop (Thr294–Asn304) can move to accommodate the DNA, allowing nicking by Can2. Clearly ssRNA as a smaller molecule could also be accommodated in this cleft. This analysis suggests that the mechanism of cA_4_-dependent activation is likely to be via allosteric structural reorganisation that opens the cleft, allowing access to DNA and RNA substrates.

## DISCUSSION

Here we show that Can2, a widely distributed CARF-superfamily protein associated with type III CRISPR systems, is a cA_4_-activated DNA nickase and RNase that can provide immunity against bacteriophage *in vivo*. Can2 is a member of the DUF1887 protein family, whose founding member is the VC1899 protein from *V. cholerae* – one of the first CARF family proteins to be studied structurally (PDB: 1XMX), although the structure has never been described in a publication. VC1899 has remained uncharacterised, and notably is an orphan CARF protein that is not associated with a type III CRISPR system. Our biochemical studies indicate that it has weak residual RNase activity that is not regulated by cA_4_, and structural studies indicate that the site where cA_4_ would bind (based on conservation with Can2) is largely composed of negatively charged and hydrophobic residues that would not be conducive to binding of a negatively charged cA_4_ molecule. Therefore, VC1899 may represent a degenerate defence enzyme that has been ‘cast adrift’ from a CRISPR system and does not retain its original function. In contrast, the Tsu and Sth Can2 orthologues are robust metal and cA_4_-dependent nucleases. Like Can1, the primary role of Can2 may be to function as a nickase in infected cells, generating single-strand ligatable nicks in supercoiled DNA to slow down phage replication (19). Structural comparisons with the EndoMS nuclease suggest that Can2 is activated upon binding cA_4_, to enable binding of nucleic acid substrates between the two nuclease domains in a position ideal for catalysis.

Whilst there are obvious differences in the cA_4_ binding site between Can2 and VC1899, the nuclease domain is conserved, and VC1899 has the key nuclease active site residues (Glu276/Asp278 in Can2 and Glu291/Asp293 in VC1899). Although VC1899 is not active *in vitro*, structural comparison with Can2 is still relevant as it may highlight differences in the active (cA_4_ bound) and inactive (no cA_4_ bound) states. Superposition of Can2 with VC1899 has an RMSD of 2.7 Å over 380 Cα atoms, which is higher than just the CARF domain (RMSD of 1.7 Å over 141 Cα atoms) or just the nuclease domain (RMSD of 2.5 Å over 186 Cα atoms), suggesting there is a difference in the orientation of the domains in relation to each other. This is indeed observed (Supplementary Figure S5B), and it is clear these differences arise in the nuclease domains rather than the CARF domains which show high structural conservation (Supplementary Figure S5A). There are differences in the position of equivalent secondary structure elements throughout the nuclease domain, with the largest being an α-helix that faces into the DNA binding site. There is an approximate ∼10 Å difference in the position of the helix, where Can2 (cA_4_ bound) has the helix from each monomer in the dimer closer than in VC1899 (no cA_4_ bound). This causes a change in an approximate helix-to-helix distance of ∼45 Å in VC1899 to ∼25 Å in Can2 (Supplementary Figure S5B). In addition, a loop originating from a different part of the nuclease domain appears to extend further into the DNA binding region in Can2 than VC1899; the caveat being this loop is partially disordered in both structures and so is evidently flexible. These differences in the position of elements of the nuclease domains likely indicate the state prior to binding of DNA, whereby Can2 has been activated and VC1899 has not. It can therefore be hypothesized that the helix and loop that face into the DNA binding site likely play a role in DNA binding and orientating the substrate to the nearby nuclease active site.

Whilst comparison of Can2 and VC1899 can suggest the effect of cA_4_ binding upon rearrangements in the nuclease domain that enable DNA binding, it is more difficult to predict (a) how cA_4_ itself binds as it is likely to require significant structural rearrangements and (b) how binding of cA_4_ leads to activation of the nuclease domain. Small angle X-ray scattering experiments and temperature factor analysis performed with Can1 suggested that the nuclease and nuclease-like domains were more mobile than the CARF domains (19). This led to a model whereby Can1 was sampling between open and closed states, and cA_4_ could bind when in the open state. Upon binding, the protein would be stabilized and ‘lock’ Can1 in the closed state. Such a model would also explain why it was difficult to get diffracting crystals for both apo Can1 and Can2. However, Can1 is unusual in that it has two CARF domains and both nuclease/nuclease-like domains in a single polypeptide, whereby Can2 is a dimer with just one CARF and nuclease domain. The question therefore remains whether the dimer is pre-formed based on interactions within the CARF domain and samples between an open and closed state as postulated for Can1, or cA_4_ binds to a monomer of Can2, which enables the dimer to form.

One interesting observation is the difference in the overall size of Can2 relative to Can1; whilst Can1 only has ∼100 residues less than the Can2 dimer, the overall dimensions of the structure indicate it is much more compressed, with the four domains tightly packed (Supplementary Figure S7B, C). Although difficult to measure with precision, Can2 is ∼15–20 Å larger if measured from the top of the nuclease domain to the bottom of the CARF domain. The cA_4_ molecule is enclosed in Can2, meaning protein residues would need to move to allow it to bind, however, there is more void space surrounding the cA_4_ molecule in Can2 than Can1. Given this relative greater distance between the cA_4_ molecule and the nuclease domain, it makes it more difficult to ascertain which residues in the nuclease domain may be involved in ‘sensing’ the binding of cA_4_ leading to activation of the nuclease domain. In Can1 there is a direct interaction between Gly550, which originated from the nuclease domain, with the cA_4_. In addition, Gln222, from the nuclease-like domain, sits above the centre of cA_4_ and makes two water-mediated hydrogen bond interactions with phosphate groups on opposite sides of the ring. There are no residues equivalent to these in Can2, which is a consequence of the less dense packing, and so there must be further subtle rearrangements of residues to enable this activation message to be transmitted.

The observation that SthCan2 can degrade both DNA and RNA is unusual but not unprecedented. Although the preferred cation is Mn^2+^, RNase activity is also supported by Mg^2+^ at a lower level. Other nucleases with this dual specificity include the tomato multifunctional nuclease TBN1, which is a member of the phospholipase C/P1 nuclease family (47), and *Serratia marcescens* endonuclease of the EndoG family (48). The latter has been used to design a synthetic conditional suicide system in *E. coli* (49), analogous to the altruistic suicide seen in abortive infection. The observation that TsuCan2 primarily degrades RNA substrates *in vitro*, with only weak DNA nicking activity observed at high temperatures, suggests that this enzyme may primarily function as a cA_4_-activated non-specific RNA degrading nuclease, analogous to the Csm6/Csx1 family of CRISPR ancillary nucleases. The rate of RNA cleavage, which exceeds 1 min^−1^ under single turnover conditions, is comparable with rate constants observed for Csm6/Csx1 family enzymes (12,22).

The demonstration that Can2 can be ‘hooked up’ with the *M. tuberculosis* type III-A CRISPR system, which normally functions via the cA_6_-activated Csm6 ribonuclease (15), emphasises the power and flexibility of type III cOA signalling, where the cyclic nucleotide second messenger and effector nuclease can be swapped without losing immunity. Frequently, multiple effectors are observed associated with one type III system (for example, the NucC and Can2 effectors present in *M. ishizawai*, shown in Figure 1). This flexibility may be another reflection of the pressure imposed by phage anti-CRISPRs (Acrs) – as for example a cA_4_-specific Acr could knock out Can2 or Csx1, but not Csm6 or NucC. It is notable that Can2 provides immunity from phage P1 infection, without causing a noticeable growth defect in host cells. This suggests that defence based on Can2 operates by slowing down phage replication, rather than an abortive infection mechanism. The ligatable nicks generated by SthCan2 may therefore preferentially target replicating phage DNA, as suggested for the Can1 enzyme (19). The observation of variable nickase activity in the Can2 orthologues may indicate that RNA cleavage is the predominant immune activity *in vivo* – placing Can2 closer to the Csx1/Csm6 family than the Can1 family. Further work is required to ascertain whether Can2 can provide similar levels of immunity against a broader range of bacteriophages, including RNA phages.

While this paper was in the final stages of revision, an independent study of the Can2 enzyme from *Treponema succinifaciens* was published. The enzyme, which was named Card1, has similar properties as a manganese and cA_4_ dependent relaxed specificity nuclease (50).

## DATA AVAILABILITY

The molecular coordinates for the Can2 protein structure are available in the Protein Databank under accession code 7BDV.

## Supplementary Material

gkab073_Supplemental_File
