# Supplementary material for: The CRISPR ancillary effector Can2 is a dual-specificity nuclease potentiating type III CRISPR defence
Source: Nucleic Acids Res. 2021 Feb 15;49(5):2777–89. doi: 10.1093/nar/gkab073 (PMC7969007; doi:10.1093/nar/gkab073)
Supplement: gkab073_Supplemental_File [file gkab073_supplemental_file.pdf]

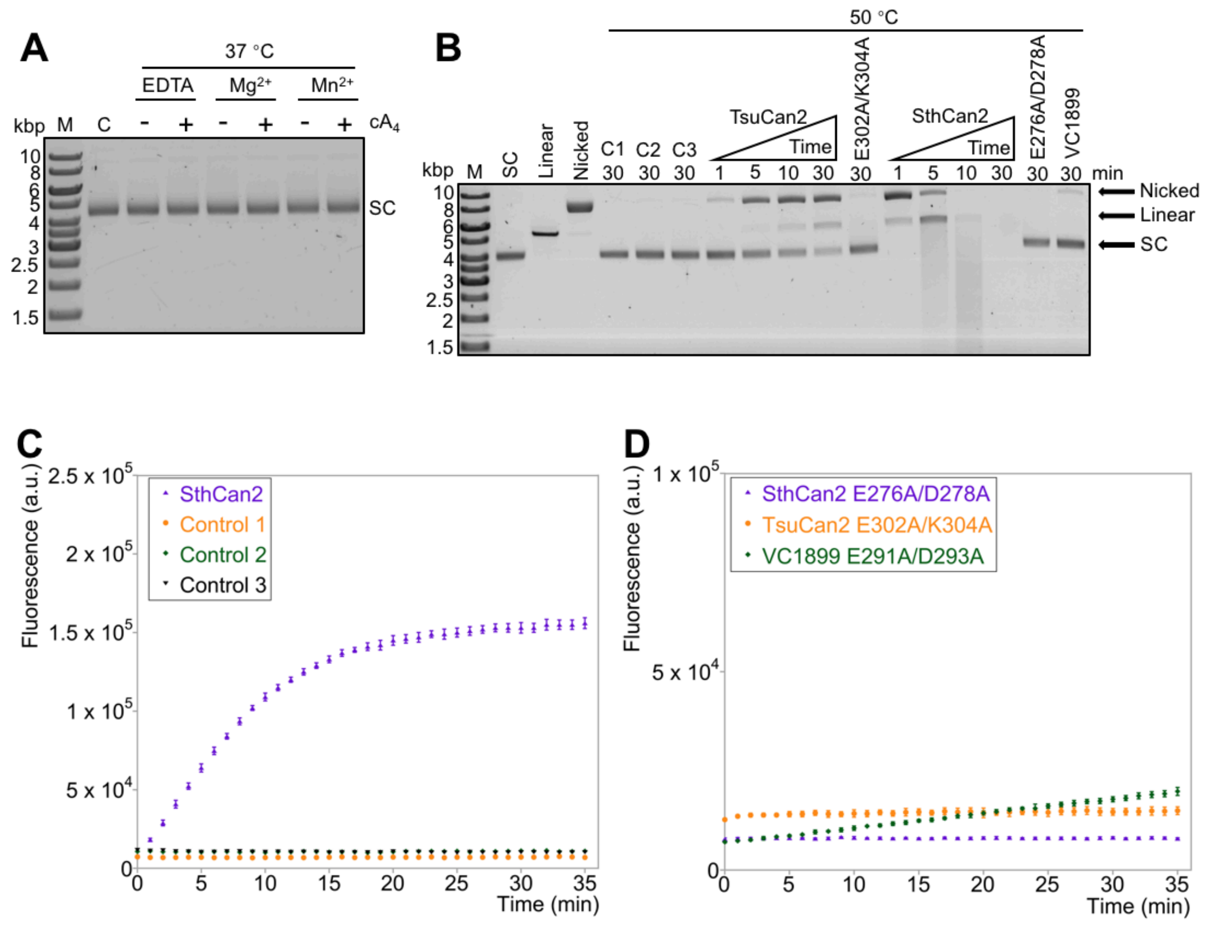

Supplementary Figure 1. scDNA and RNaseAlert substrates cleaved by wild-type SthCan2 and its homologues TsuCan2 and VC1899. A. scDNA cleavage by VC1899 at 37 °C. scDNA (1.8 nM) was incubated with VC1899 (500 nM dimer) under the same conditions described in Figure 2A for 30 min supplemented with cA_4 (1 µM) and $MgCl_2$ (5 mM) or $MnCl_2$ (5 mM). No scDNA cleavage was observed. **B.** scDNA cleavage by TsuCan2, SthCan2 and VC1899 at 50 °C. scDNA (1.8 nM) was incubated with enzymes (500 nM dimer) under the same conditions described in Figure 2A for the time indicated supplemented with cA_4 (1 µM) and $MnCl_2$ (5 mM). scDNA was degraded rapidly by SthCan2. Most of scDNA was nicked and some scDNA was linearized by TsuCan2 after 30 min. No activity was observed for their nuclease variants. No activity was observed for VC1899. Standards corresponding to supercoiled (SC), linear and nicked plasmid are shown after the marker (M) lane. Control lanes C1, C2 and C3 show the reactions incubated with TsuCan2 for 30 min without protein, $MnCl_2$ and cA_4, respectively. **C.** Plot of fluorescent signals emitted by RNaseAlert substrates when they were cleaved by SthCan2. RNaseAlert substrates (30 nM) were incubated with the enzymes (500 nM dimer) under the same conditions described in Figure 2C at 37 °C. Control1, Control2 and Control3 represent the reactions incubated without protein, $MnCl_2$ and cA_4, respectively. Values and error bars for SthCan2 represent the mean of triplicate experiments and the standard deviation. **D.** Plot of fluorescent signals emitted by RNaseAlert substrates when they were cleaved by nuclease variants of SthCan2, TsuCan2 and VC1899. RNaseAlert substrates (30 nM) were incubated with the enzymes (500 nM dimer) under the same conditions. Very little activity was observed. Values and error bars represent the mean of triplicate experiments and the standard deviation.

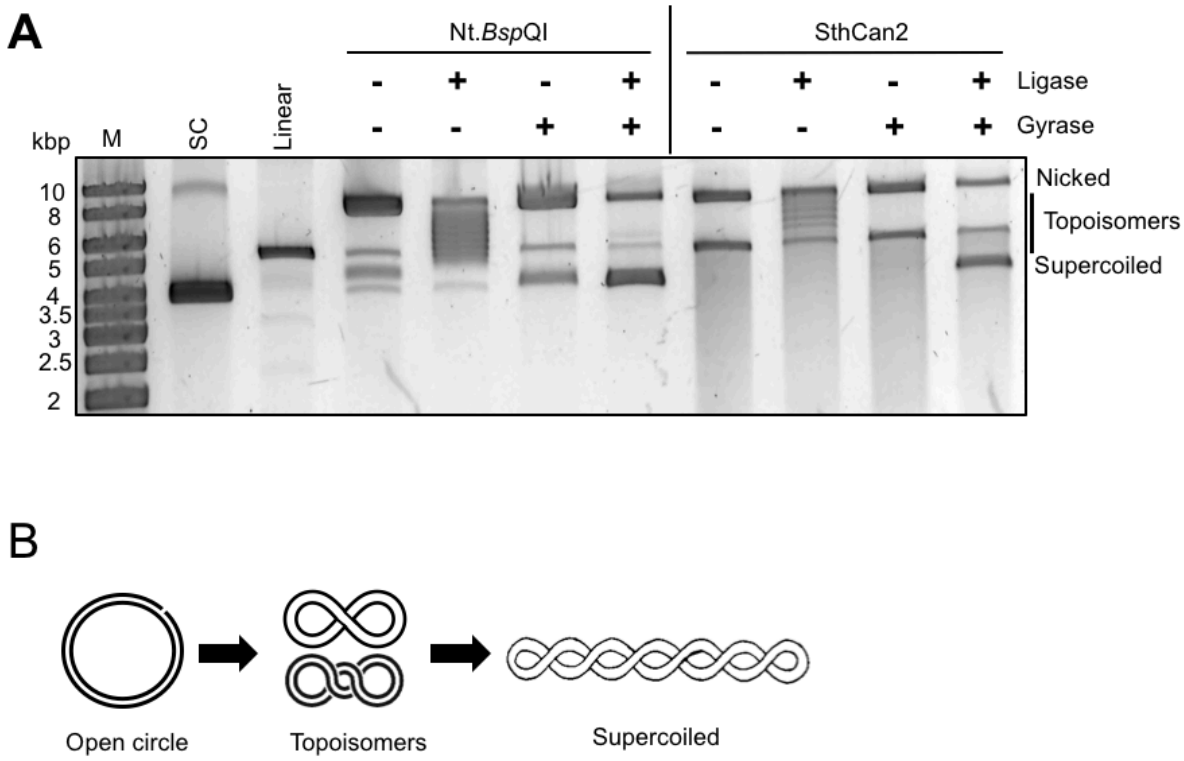

Supplementary Figure 2. Ligation and supercoiling of DNA nicked by SthCan2. A. Open circle DNA was incubated with DNA ligase and gyrase as detailed in the Methods. DNA ligase ligated the nicked site in the plasmid, generating a range of topoisomers. DNA gyrase restored the structure of the supercoiled DNA. **B.** Schematic of the plasmid structure following incubation with DNA ligase and gyrase.

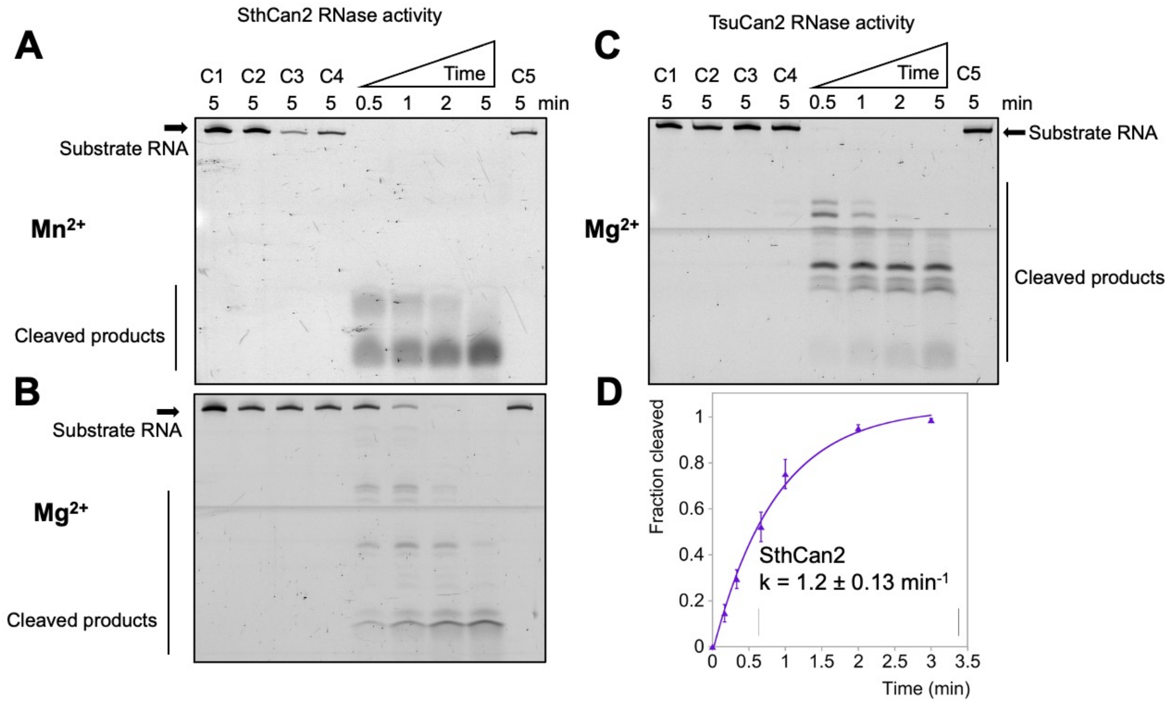

Supplementary Figure 3. Can2 is activated by cA$_4$ to degrade 5'-FAM labelled ssRNA. A. Denaturing PAGE analysis of 5'-FAM labelled ssRNA (30 nM) cleavage by SthCan2 (500 nM dimer) and E276A/D278A variant (500 nM dimer). The reaction was carried out at 50 °C for 0.5, 1, 2 and 5 min with wild-type SthCan2 and 5 min with E276A/D278A (lane C5) in reaction buffer supplemented with 1 μM cA$_4$ and 5 mM $MnCl_2$. Control lane C1 represents incubation of RNA only in the reaction buffer at 50 °C for 5 min. C2, C3 and C4 refer to the reactions incubated for 5 min without protein, $MnCl_2$ and cA$_4$, respectively. Substrate and product are indicated alongside the gel. **B.** Denaturing PAGE analysis of ssRNA substrate cleavage in the presence of $MgCl_2$. All reactions were carried out under same conditions as in part A in the presence of 5 mM $MgCl_2$ instead of $MnCl_2$. Control lane C3 refer to the reaction without $MgCl_2$. **C.** Denaturing PAGE analysis of ssRNA substrate cleavage by TsuCan2 (500 nM dimer) and E302A/K304A variant (500 nM dimer; lane C5)) in the presence of $MgCl_2$. All reactions were carried out under same conditions as in part B at 37 °C. Control lanes C1-C4 are as in part B. **D.** Single-turnover kinetic analysis of ssRNA cleavage by SthCan2 in the presence of $MgCl_2$. Reactions were under the same conditions as in part B and stopped at 10 s, 20 s, 40 s, 1 min, 2 min and 3 min. The rate constant was 1.2 ± 0.13 min^{-1}. Values and error bars represent the mean of triplicate experiments and the standard deviation.

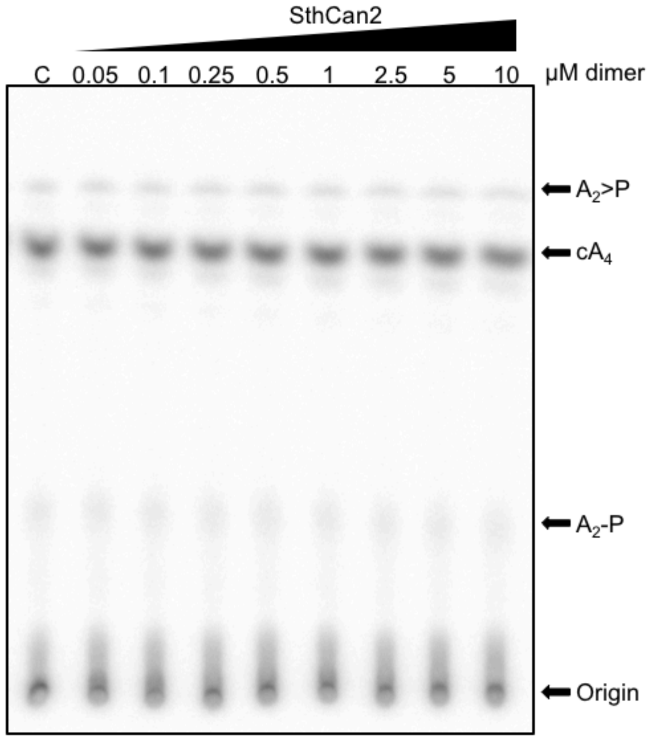

Supplementary Figure 4. SthCan2 does not degrade the cA_4 activator. ^{32}P labelled cA_4 was incubated with SthCan2 at different concentrations, designated in the figure. The control was carried out by incubating radiolabelled cA_4 in buffer only under the same conditions. Samples were loaded onto thin-layer chromatography (TLC) plates and visualized by phosphor imaging. Origin refers to loading spots. Possible cA_4 cleavage products are indicated.

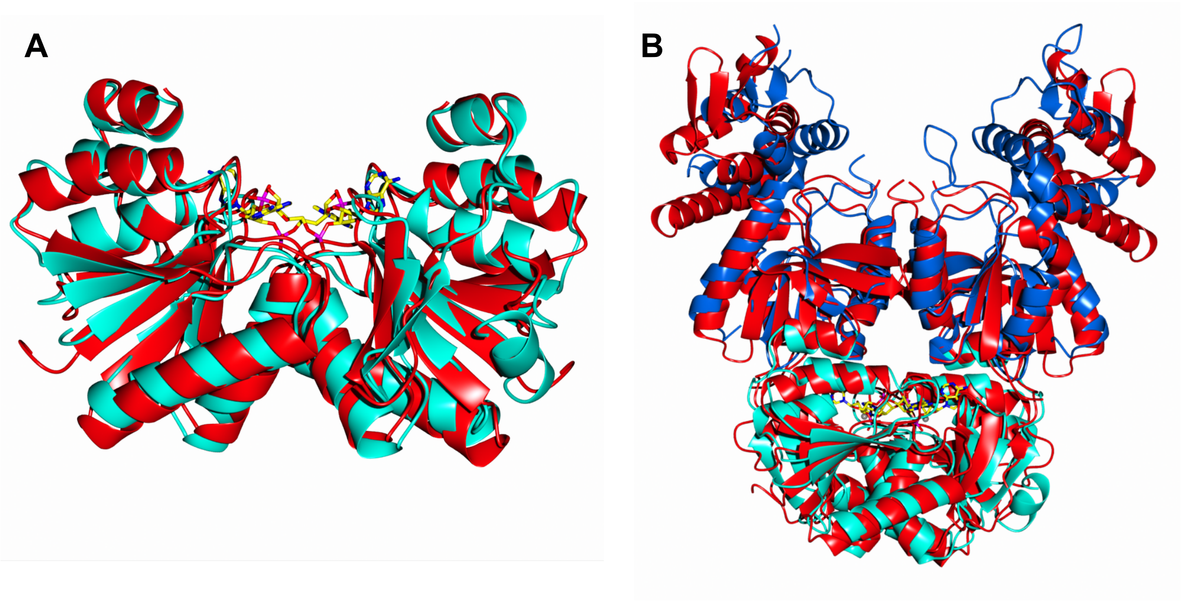

Supplementary Figure 5. Structural comparison of SthCan2 and VC1899. A. Superimposition of the Can2 (cyan) and VC1899 CARF domains (red; PDB:1XMX) in cartoon representation (RMSD of 2.1 Å over 137 Cα atoms for the Can2 monomer). The molecule of cA_4 bound at the CARF dimer interface is shown in stick representation (carbon in yellow, oxygen in red, nitrogen in blue, phosphate in magenta). **B.** Superimposition of Can2 (CARF domains in cyan and nuclease domains in blue) with VC1899 (red; PDB:1XMX) in cartoon representation, with cA_4 bound (yellow).

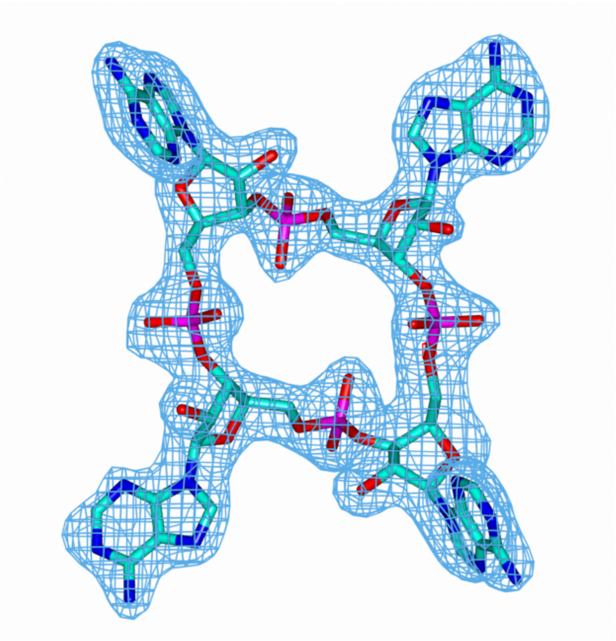

Supplementary Figure 6. Electron density map present in the SthCan2 CARF domain into which cA_4 was modelled. The cA_4 molecule in stick representation (carbon in cyan, oxygen in red, nitrogen in blue, phosphate in magenta) with the maximum likelihood/σ_A weighted F_{obs} - F_{calc} electron density map contoured at ~3.0 sigma (0.28 e/Å^3) shown in blue.

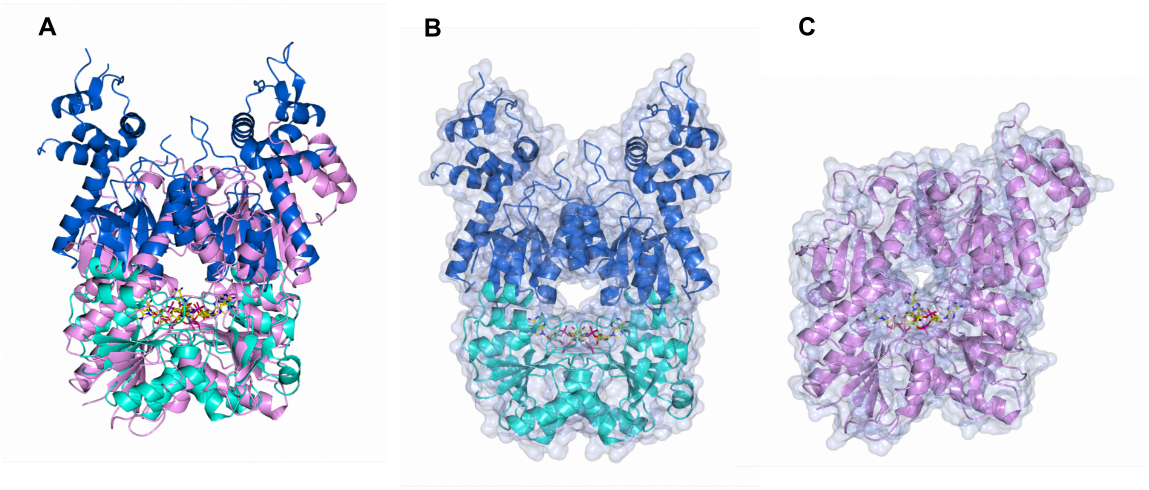

Supplementary Figure 7. Structural comparison of SthCan2 and Can1. A. Superimposition of Can2 (CARF domains in cyan and nuclease/nuclease-like domains in blue) and Can1 (pink; PDB:6SCE) in cartoon representation (RMSD of 4.0 Å over 614 Cα atoms for the Can2 dimer). The molecule of cA_4 bound at the CARF dimer interface is shown in stick representation for both Can2 and Can1 (carbon in yellow, oxygen in red, nitrogen in blue, phosphate in magenta). **B.** Surface representation of Can2 (CARF domains in cyan and nuclease domains in blue) with cA_4 in yellow sticks. **C.** Surface representation of Can1 (pink) with cA_4 (PDB:6SCE) in yellow sticks. The three panels are shown in an identical orientation.

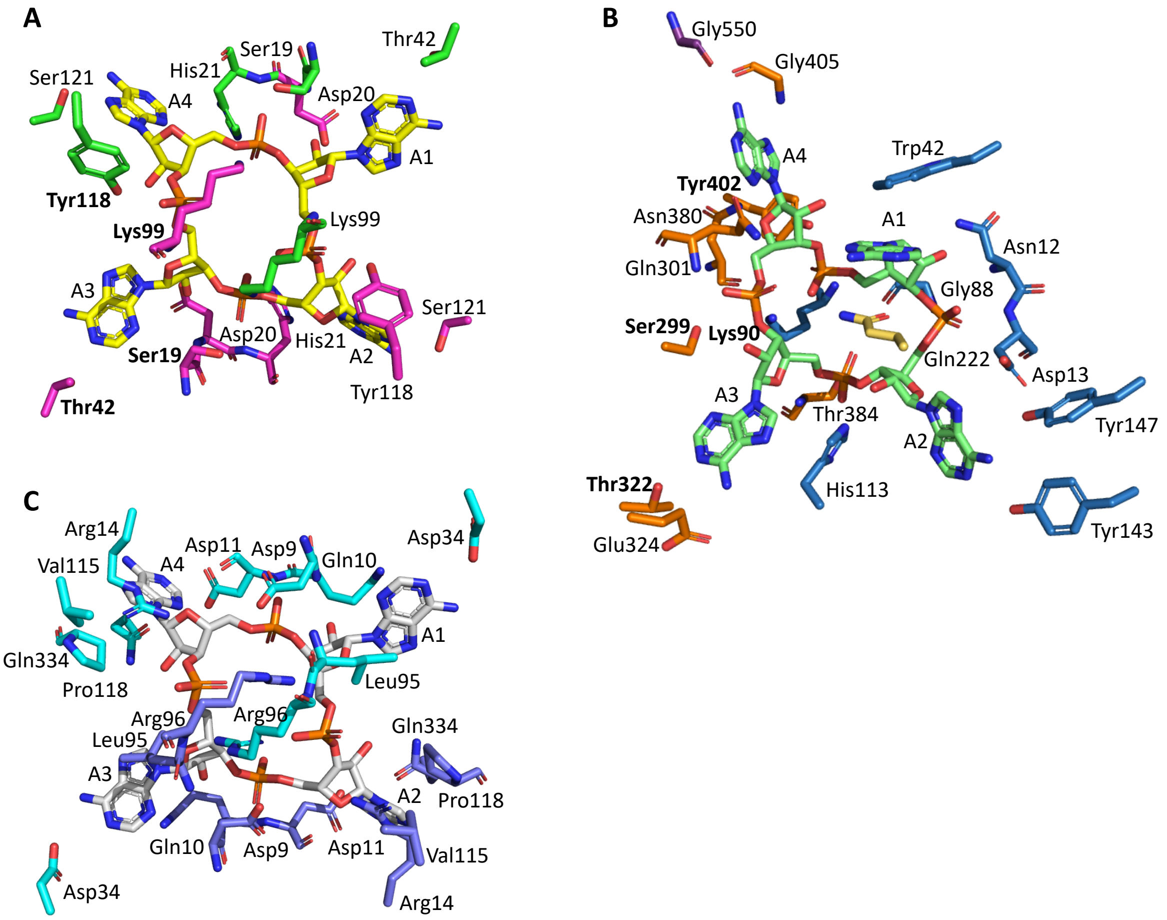

Supplementary Figure 8. Structural comparison of cA_4 bound to Can2, VC1899 and Can1. A. Stick representation of cA_4 (carbon in yellow, nitrogen in blue, oxygen in red, phosphorus in orange) in complex with the Can2 CARF domain dimer (each monomer in the dimer is coloured green or magenta). **B.** Stick representation of cA_4 (green) in complex with the Can1 CARF domains (each CARF domain is coloured orange or blue; PDB 6SCE). **C.** Stick representation of cA_4 (grey) modelled (based on the overlap of protein residues of VC1899 with Can2) in complex with the VC1899 CARF domain dimer (each monomer in the dimer is coloured cyan or mauve; PDB 1XMX). Each AMP is numbered A1-A4. Each panel is shown in the same orientation. The residue numbers in bold in panels A and B refer to the residues that are structurally conserved in Can1 and Can2 (ie. they make the equivalent interactions).

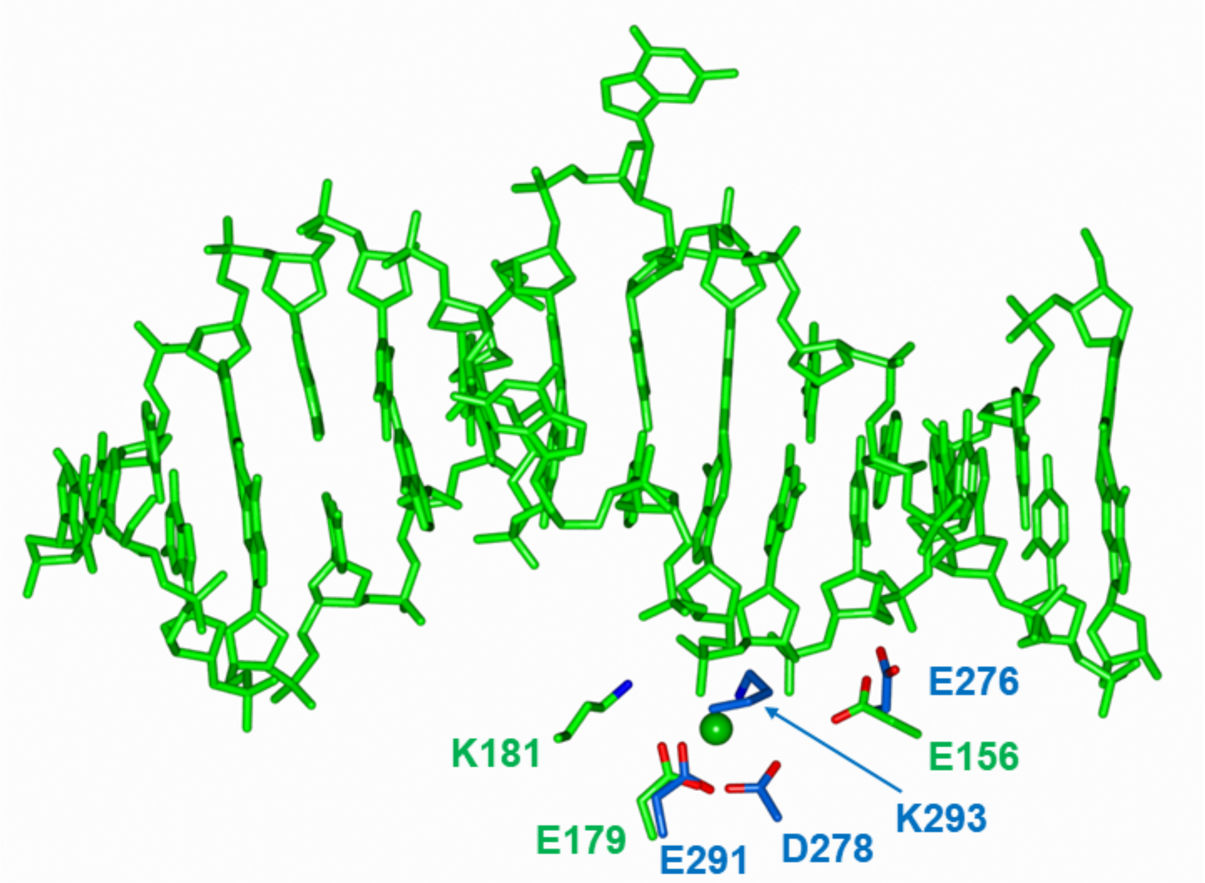

Supplementary Figure 9. Close-up view of the active sites of SthCan2 and EndoMS in complex with dsDNA. Superimposition of the nuclease domain of Can2 (blue) and the nuclease domain of EndoMS in complex with dsDNA (green; PDB: 5GKE), shown in stick representation. A magnesium ion present in the EndoMS active site is shown as a green sphere. The residue in EndoMS (Asp158) equivalent to Asp278 in Can2 was mutated in order to trap the complex with dsDNA, and therefore is not shown.

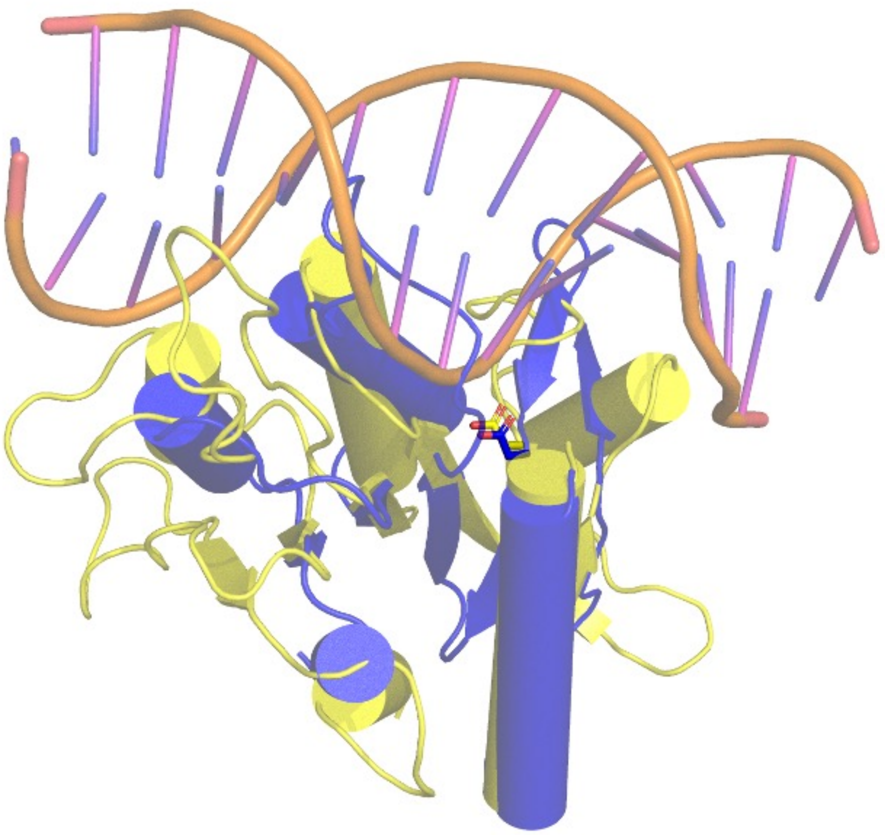

Supplementary Figure 10. Comparison of the nuclease fold of Can2 and *Age*I. Can2 is shown in blue and *Age*I (PDB 5DWA) in yellow, with the DNA present in the *Age*I structure shown in cartoon form. The key secondary structure elements and key catalytic residue D142 (*Age*I) / D278 (Can2) superimpose well. The overall RMSD is 3.0 Å over 111 Cα atoms, according to DALI.

Supplementary Table 1. Sequence of synthetic genes

Name	Sequences 5' → 3'
SthCan2	CGCGCCATGGCTCGCTTAGACGACTTATTTATTATTCATGACACTTACGTT<br>TGTTTGTTAAGTGATCACCTTTTGCCTAACGTTATCCCAGTAATTCAGGCT<br>CCCCCGCAACGTGTGATTTTACTTTACACCCCAAACAATAAAGAACGCGT<br>CCAGCGCTTCCGTCAGGCTACGGAAAGCGTGCCTACAGAGATTATTGAA<br>AAACAGGTTCACCCATACCAGTACGCACAAACTCAGCGCATCTGTGATGA<br>GATCCTTGAGCAGTTTCCAAACGCCATCCTTAACGTAACAGGGGGAACTA<br>AAATTATGGCTTTAGCGGCGTTCGACCGTTTCCGCCATAATCACCGCCCT<br>ATTATCTATGTTGACTCCGATAGCCAGCGTATCTTATATCTTCACAATGGA<br>GAGTCGGAGCGCCTGGGGGACCCCTTGACGGTGAAACAGTACTTAGCTT<br>GTTACGGGTTTAAAGCCGATAATATCAACCGCCAGGACAATCTGCCCAAA<br>ACTTGGCGCGAAGTAGAAGATCTTTTTGCGCAGAACAGCACCAAGTGGC<br>AAAACCAGCTTGGACGCTTGAATTGGATCGCGGCACAGCAGCAACCTATT<br>TTCACGCTGCAGACTGGAGAGTTGCAGGACTTACTTCTGAAGGCGAACTT<br>GATTAAACCCGCGGAGGCAAAAAACGCTGGTTTTCAATTTACCTCAGATC<br>AGGCCCGTCAATTTATCAATGGTGGCTGGTTTGAACACTATGTATACTCG<br>TTGTTACGTCAGATTTCTGCTCAATACCCAATTAAGAACCTGACTAAGAAT<br>ATCGAAATTAGTAACGATAGTGTCTCCAATGAACTGGACGTCGTTTTTTA<br>TACCACAATAAGCTGCACGTCATCGAGTGCAAAACACGTCATTTCACGGC<br>TGATGGAAAGATCAACCCGATGGAGACGATTTACAAGATTGACAGCGTCA<br>CAAACCGTGTGGCCGGAATCAAGGGAAAATCTATGTTCGCTAGTTATTAC<br>CCATTAACTCAAGCAGCCAAGAAGCGTTGTTTGAATAACTCTATTTATGTT<br>TCGGACCAGCCTAGTCAACTGCACCATCAATTAATCAAATGGATTAACGC<br>GTGAGGATCCCCGG
TsuCan2	CGCGCCATGGCGCGCTATCAAACTCATGTATATCTGGTGTCTGATCAAGC<br>AACCCCAAACCTGACCCCCGCCCTTGATCCCGATATCCGCCCCGAACGT<br>GCCGTTTTGGCGGTGACGCCTCAAGCCGAGCACCAGGCGCGCTGGCTT<br>ACAACAGTCTTAGAGCGCCACGGTGTCCACTGTGAACGTTTGTCCCTGG<br>ATGATGCTTATGACTTGGACTCCTTACGTCAATCGTTTCGCAGTTATTTGC<br>AGCGTTGTACCGAGCCGGTGGCTGTGAATGTCTCAGGCGGGAGTAAACC<br>AATGAGTATTGCTGCGTTTGAAGTCTTCGCGCATGCCGATCAGGGGGTCT<br>TTTATGTAAACCCCCGCACGGACACTTTAGACTGGTTACATCCCACGGGC<br>ATCCCTGCTCAAGCTATTGCTGATCGTGTAGGGCTTGAAGATTTTTTGGA<br>GGCTCACGGGGCTTCTGTCCATCACCTGTCTCGCCAGGCGGCTCCTGCA<br>TCGCGCCTTGCTTTGTACGAAGAAATTGTGCGTTTGCGTGGCCGCTGGA<br>AAAAACATGGCTCTATTGGCTTGTTGAACCGTTATGCACAGGGTGCTCGT<br>GAAACGCTTCGCTCTGAAGAAATTTCTCCAGAACATCAACGTCTTTTAAAT<br>GAGCTTCCAGGCCTGTTCGCGGAACAGGGTCTTCTTTCATGGGAGGGAA<br>CACGTCTTGTCTTTCCAGATGAACAAGCTCGTCGTTTGGTGAACGGGGTA<br>TGGCTGGAGGAATATCTGTTTAGTCGCTTACTTGCCTTGAAAGACAGTTT<br>ACCAGCGATGCAAGATCTGGCAGCCAACGTGGTGGTCCGTCGCAATACA<br>GACAACGGGTATGTTCAGAATGAGCTGGACGTGGCGTTGCTGTTGGACA<br>ATCGCTTATGGGTACTGGAATGCAAGGCTTCGAATACCTTCGCACCTCAG<br>AATCGTGTAGATCAGGCTACCCGTCAGTCTTTGTACAAACTTGAAGCATT<br>ATTGACCCCCTTGGGGGGAATTTTGGCCCGCGGTATGTTAGTGAACGTA

	CTGCCGATCGGGGTAAATGATCAGCGTCGTATTGACAACAACCCGCGCT<br>TATGTGCGTTGACTTATCGTGACTTCGATGACTTGGACCGTCACTTACAT<br>GCTCGCCTTGCAGCACAA TGAGGATCCCCGG
VC1899	CGCGCCATGGCAATCCATGTCGGGATCATTGACCAAGATCCCGTGCGTC<br>TGGTGACACCACTTTTAGACCACCGCACGGTAAGTCGTCACATTATCTTT<br>ATCGGTGATCACACACAGACTGTTATCTACCAACGTCTGTCAGATGTTTT<br>GAATAAGCGCAATATCTCTACTGACTTTTTCGAAATTCCGGCGGGCAGTA<br>ACACCTCAGCTATTAAATCTGCCATTCGTGAGCTGGCGGAAACATTGAAA<br>GCGCGCGGTGAGGAAGTTAAGTTCAATGCATCCTGCGGTCTTCGCCATC<br>GCCTTTTAAGCGCATACGAAGTGTTTCGTAGTTACCACTGGCCGATCTTC<br>GTAGTGGAACCCAATTCCGACTGTTTATGCTGGCTTTATCCCGAAGGTAA<br>TAATGATACTCAGGTCCAAGACCGCATTACAATCGCGGACTACTTAACGA<br>TTTTCGGGGCCCGTGGTGAGTTCAACGAACATCAGTTAAGCCCACAGTTA<br>GACCAGCAACTTTATCAGTTAGGTGAGCGCTGGGCTAGTAACGCATTGG<br>AATTGGGTCCTGGCTTAGCCACGCTTAACTACTTAGCCACAACGTGCCGC<br>AAGGAACAAAAATTAGATGTGGAACTGTCCGACAAGCAACAAGGATACCG<br>TGAGTTAAACCTGTTATTGTCTGACCTGGTTGAGGCGAAGATCGCGAGCT<br>ACGAGAACGGGATTCTGACTTTTATTAATGAAGAAGCACGCCGCTTCGCT<br>AATGGCGAGTGGTTAGAAACTCTTGTCCATTCTACGGTGAAACAGATCCA<br>AGACGACATGCCAACTATCCAAGATCGTTCACTGAACGTGCAAGTGTACC<br>GCCAGTTGGGCGAACGCGAAGTACGCAATGAGCTTGACGTAGCAACCGT<br>CGTAAACAACAAGCTGCATATTATTGAATGCAAAACGAAAGGAATGCGTG<br>ACGACGGCGACGACACGTTGTACAAGTTAGAATCGCTTCGTGACTTGCTT<br>GGAGGTTTACAAGCGCGCGCGATGCTTGTATCCTTTCGCCCCCTTCGTC<br>ATAATGATATCACTCGTGCAGAGGACCTGGGACTGGCGCTGATCGGTCC<br>TGATGAGTTAAAAGACTTAAAGACCCATCTTACGCAATGGTTTAAGGCGG<br>CCGGAGGAAATTGAGGATCCCCGG

Supplementary Table 2. Oligonucleotides and primers used for mutagenesis

Name	Sequences 5' → 3'	Note
SthCan2 E276A/D278Af	GTCTCCAATGCACTGGCCGTCGTTTTTTTATAC	Mutagenesis for SthCan2
SthCan2 E276A/D278Ar	GTATAAAAAAACGACGGCCAGTGCATTGGAGAC	Mutagenesis for SthCan2
TsuCan2 E302A/K304Af	CGCTTATGGGTACTGGCATGCGCGGCTTCGAATACCTTCGC	Mutagenesis for TsuCan2
TsuCan2 E302A/K304Ar	GCGAAGGTATTCGAAGCCGCGCATGCCAGTACCCATAAGCG	Mutagenesis for TsuCan2
VC1899 E291A/D293Af	CGCGAAGTACGCAATGCGCTTGCCGTAGCAACCGTCGT	Mutagenesis for VC1899
VC1899 E291A/D293Ar	ACGACGGTTGCTACGGCAAGCGCATTGCGTACTTCGCG	Mutagenesis for VC1899
5'-FAM labelled ssRNA	AUUGAAAGACCAUACCCAACUUCUAACAACGUCGUUCUUAACAACGGAUUAAUCCCAAAA	Substrate ssRNA
Lpa target	ATTCGTGAGTGATTTATTTCCATGAAGTGGCGTCCCT	*lpa*-targeting spacer

Supplementary Table 3. Data collection and refinement statistics of Can2 in complex with cA_4 (PDB: 7BDV)

	Can2 + cA_4
**Data collection**	
Wavelength (Å)	0.9790
Space group	P 2_1 2_1 2_1
Cell dimensions	
a, *b*, *c* (Å)	75.86, 85.97, 237.91
α, β, λ (°)	90, 90, 90
Resolution (Å)	51.32 – 2.02 (2.07 – 2.02) *
Total number of observations	3638547
Total number of unique observations	102945
R_{merge}	0.18 (2.17)
$I / \sigma I$	16.0 (2.1)
Completeness (%)	100.0 (100.0)
Redundancy	35.3 (39.0)
CC(1/2)	0.99 (0.57)
Anomalous completeness (%)	100.0 (100.0)
Anomalous multiplicity	18.2 (19.8)
Mid-slope of anomalous normal probability	1.081
**Refinement**	
Resolution (Å)	51.37 – 2.02
No. reflections	97728
R_{work} / R_{free}	0.24 / 0.29
No. atoms	10989
Protein	10540
Ligand/ion	176
Water	273
B-factors (Å^3)	
Protein	53.2
Ligand	37.7
Water	49.6
RMSD**	
Bond lengths (Å)	0.008
Bond angles (°)	1.52

* Values in parentheses are for the high resolution shell.

** RMSD, root mean square deviation.
